# *Drosophila* FIT is a protein-specific satiety hormone essential for feeding control

**DOI:** 10.1038/ncomms14161

**Published:** 2017-01-19

**Authors:** Jinghan Sun, Chang Liu, Xiaobing Bai, Xiaoting Li, Jingyun Li, Zhiping Zhang, Yunpeng Zhang, Jing Guo, Yan Li

**Affiliations:** 1 State Key Laboratory of Brain and Cognitive Science, Institute of Biophysics, Chinese Academy of Sciences, Beijing 100101, China; 2 University of Chinese Academy of Sciences, Beijing 100049, China; 3 Sino-Danish Center for Education and Research, Beijing 100190, China

## Abstract

Protein homeostasis is critical for health and lifespan of animals. However, the mechanisms for controlling protein feeding remain poorly understood. Here we report that in *Drosophila*, protein intake-induced feeding inhibition (PIFI) is specific to protein-containing food, and this effect is mediated by a fat body (FB) peptide named female-specific independent of transformer (FIT). Upon consumption of protein food, FIT expression is greatly elevated. Secreted FIT peptide in the fly haemolymph conveys this metabolic message to the brain, thereby promoting the release of *Drosophila* insulin-like peptide 2 (DILP2) and suppressing further protein intake. Interestingly, *Fit* is a sexually dimorphic gene, and consequently protein consumption-induced insulin release, as well as protein feeding behaviour, are also dimorphic between sexes. Thus, our findings reveal a protein-specific satiety hormone, providing important insights into the complex regulation of feeding decision, as well as the sexual dimorphism in feeding behaviour.

## Abstract (web summary)

Protein feeding is known to induce strong inhibition on further food intake, though the underlying mechanisms remain poorly understood. Here, the authors identify a protein-specific satiety hormone in *Drosophila*, and show that it suppresses feeding via promoting DILP2 release in the central nervous system.

Nutritional homeostasis is crucial for animal survival and health, and nutrition states must therefore be tightly monitored by the nervous system, which in turn orchestrates feeding behaviour^1^,^2^,^3^,^4^,^5^. Dietary protein provides not only resources for energy, but also materials for protein synthesis, and either less or excess in protein consumption results in severe developmental defects or health problems^6^,^7^. Accordingly, dietary protein plays important roles in the proper regulation of feeding behaviour^1^,^8^. For example, animals reject food void of essential amino acids, and studies in both mice and flies indicate that the deficiency in essential amino acids is directly detected by the central nervous system (CNS), namely through a cell-based mechanism utilizing the evolutionary conserved kinase GC nonderepressing 2 (refs 9, 10). Moreover, total food intake is increased or decreased when the protein content in food is low or high, respectively^8^. Among the three macronutrients, protein exerts the greatest inhibitory effect in feeding regulation^1^,^11^. Together, these phenomena suggest that there are protein-specific satiety signals; however, their identification awaits elucidation.

In mammals, protein consumption triggers multiple pre- and post-absorptive signals, and major afferent signals for the brain are neural signals mediated by vagus, and hormone signals mediated by peripheral peptides circulating in the blood^1^. Particularly, anorexigenic gut peptides, such as Cholecystokinin, Peptide Y, and Glucagon-like peptide-1, are proposed to mediate the satiating effect of dietary protein^1^. Nevertheless, none of these neural and hormone signals are found to be protein-specific, and they are deemed to convey information about the energy status of food consumption. Therefore, it remains to be elucidated as to why protein food possesses the highest feeding suppression effect. Long-term ingestion of protein is thought to be one reason; however, this concept is still not widely accepted^6^.

Strong feeding suppression as a result of high-level protein consumption is also observed in *Drosophila* larvae^12^. In addition, a recent study on olfactory learning and memory showed that fly larvae are capable of grading the nutrition value of amino acids (AAs) and sugar, and moreover, are able to distinguish between these two type of nutrients^13^. In adult flies, protein deprivation causes a significant shift in food preference to protein food^14^. These observations suggest that specific signals are present reflect the internal AA state and act on the CNS to control feeding. In *Drosophila*, the fat body (FB) is the functional homologue of mammalian adipose tissue, and the AA transporter *Slimfast* (*Slif)* and the mechanistic target of rapamycin (mTOR) pathway play critical functions in the FB for monitoring the internal AA state in larval development^15^. The nutrition signals in the FB are assumed to be conveyed to the CNS by secreted factors in the larval haemolymph^16^. The signal of sugar and fat states was found to be mediated by cytokine unpaired 2 (Upd2), which regulates brain insulin signalling remotely^17^. Two recent studies identified growth-blocking peptides (GBP1 and GBP2) and Stunted (Sun) as AA-responding peptides secreted from the FB. These peptides also control the systemic growth of larvae by promoting the secretion of *Drosophila* insulin-like peptides (DILPs)^18^,^19^. Nevertheless, AA-specific factors responsible for eliciting a behavioural change in feeding have so far eluded identification.

Insulin is one of the most important hormones for controlling energy homeostasis and glucose metabolism^20^. Furthermore, CNS insulin signalling is known as the key negative regulator for food intake and body weight highly conserved throughout evolution^21^. High concentrations of insulin have been found in mammal brains, together with insulin receptors (InRs) widely distributed across different brain regions^22^. DILPs are highly conserved with the mammalian homologues^23^. Different from mammals, DILPs are mainly produced by a group of secretory neurons called insulin-producing cells (IPCs), within the pars intercerebras (PI) region in the fly brain. This region contains various neurosecretory cells and is considered to execute similar function of the mammalian hypothalamus^24^. Moreover, it has been shown that insulin signalling in the fly brain is also essential for controlling feeding behaviour in both larvae and adults^25^,^26^.

In the study presented here, we investigated protein-specific satiety signals in *Drosophila* and identified the FB peptide female-specific independent of transformer (FIT) as a messenger factor for such signal. FIT expression was found to be elevated specifically upon protein consumption, and in turn high levels of FIT suppressed feeding behaviour specific for protein food. Furthermore, we demonstrated that FIT is a secreted factor that exerts its function by promoting DILP2 release in the brain.

## Results

### *Fit* expression is elevated selectively by protein-intake

**Figure 1:**
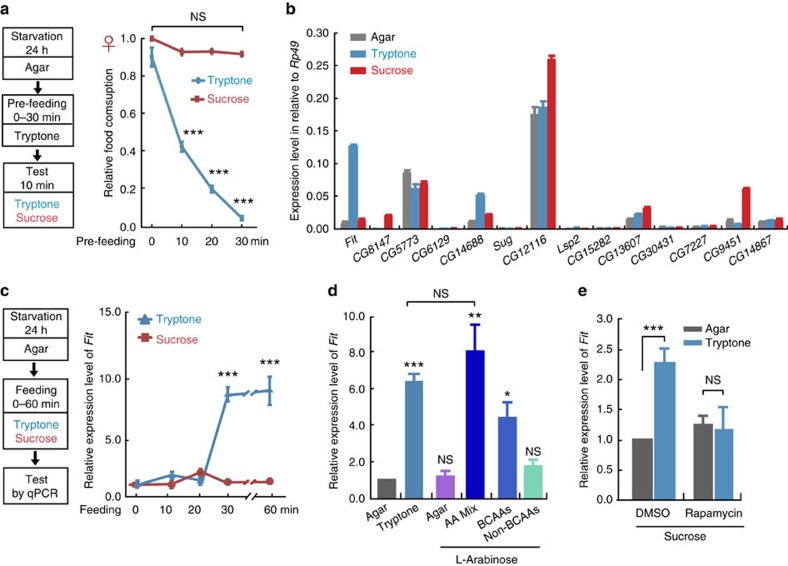
***Fit*** **expression specifically responds to protein intake.** (**a**) Along the increase of tryptone pre-feeding time, tryptone intake, but not sucrose intake, decreased significantly in WT flies. *n*=6–15. P (Food*Time)=1.03E-23 (two-way ANOVA, Bonferroni test). (**b**) Gene expression levels (normalized to *Rp49*) in flies after 24 h-starvation, or after sucrose or tryptone feeding for 30 min following starvation. Among the 14 genes tested, *Fit* showed a dramatic increase of gene expression after tryptone feeding, not, however, after sucrose feeding. *n*=3. (**c**) *Fit* expression was significant increased after 30 min of tryptone feeding, while remained unchanged after sucrose feeding. *n*=3. P (Food*Time)=8.17E-8 (two-way ANOVA, Bonferroni test). (**d**) *Fit* expression increased at comparable levels after 30 min feeding of tryptone and AA mix. BCAAs showed bigger effect than non-BCAAs in promoting *Fit* expression. *n*=3. One-way ANOVA, LSD's *post hoc* test. (**e**) Tryptone feeding-induced *Fit* expression was blocked upon Rapamycin treatment. *n*=3. P (Food*Drug)=6.02E-4 (two-way ANOVA, Bonferroni test). L-Arabinose and sucrose served as the sweeteners in **d** and **e**, respectively. *P<0.05; **P<0.01; ***P<0.001. NS indicates no statistical significance. The data are mean±s.e.m.

Among the different macronutrients, protein is recognized to be the strongest inhibitor for feeding. To determine whether there are protein-specific satiety signals, we designed a pre-feeding paradigm. Following a starvation for 24 h, flies were pre-fed for 0–30 min with tryptone as protein food, and the satiety was then evaluated by a subsequent test of feeding with tryptone or sucrose for 10 min (Fig. 1a). When tested with tryptone, the food intake gradually decreased with an increase in pre-feeding time and reached almost zero in flies that had been pre-fed with tryptone for 30 min. In contrast, when tested with sucrose as sugar food, the food intake remained unchanged following tryptone pre-feeding for 30 min (Fig. 1a). Additional experiments showed that the sugar food we used was nourishing enough for this type of test. This conclusion was based on the fact that when flies were both pre-fed and tested with sucrose, we observed strong feeding suppression after 30 min pre-feeding (Supplementary Fig. 1). Together, these findings indicated that protein consumption-induced satiety was transduced into feeding inhibition in a nutrient-specific manner.

On the basis of these observations, we assumed that there are molecules specifically responding to the consumption of protein, but not to other nutrients, such as sugar. A previous high-throughput study showed that the expression levels of a number of genes changed following starvation^27^. Among the 14 genes exhibiting the most significant changes in that study, we found that after 30 min-tryptone feeding, one gene, namely *Female-specific independent of transformer* (*Fit*), was dramatically up-regulated, with no changes observed following sucrose or lipid consumption, relative to starvation control groups (Fig. 1b). In addition to *Rp49*, which showed no expression change upon feeding, two genes, *Dp* and *Actin 5C*, were used as the internal control in the quantitative PCR (qPCR) experiments to determine FIT expression (Supplementary Fig. 2a–c). With an increase in tryptone concentration, *Fit* expression levels were gradually increased and reached the platform at the concentration of 1.7% (Supplementary Fig. 2d). Thus, we used 1.7% tryptone in all experiments except those were specified. We next analysed the time response curve of *Fit* expression and found that, while it remained at low levels during the first 20 min, *Fit* levels were up-regulated by ∼8-fold 30 min post tryptone-feeding (Fig. 1c). Together, these results suggested that *Fit* expression was induced by a post-digestive signal, which is likely to be the increased levels of internal AAs.

Tryptone consists of a mixture of various AAs, as well as a small amount of other elements. We therefore tested whether AAs themselves promoted *Fit* expression. We mixed all known AAs at the concentrations present in tryptone (Supplementary Table 1), and found that this AA mix strongly induced *Fit* expression, to levels similar to those triggered by tryptone feeding (Fig. 1d). More specifically, we found that at equal concentrations, branched-chain AAs (BCAA) had a stronger effect than non-BCAA (Fig. 1d and Supplementary Fig. 2e). TOR pathway is the major pathway of AA sensing, with BCAA shown earlier to activate the TOR pathway more efficiently than non-BCAA (ref. 28), suggesting that the TOR pathway was involved in *Fit* activation. Therefore, we utilized Rapamycin, a specific inhibitor of TOR to transiently block the TOR pathway. Remarkably, after tryptone feeding, *Fit* expression was no longer elevated in the Rapamycin-treated group, whereas it was successfully induced in the dimethylsulphoxide (DMSO) control group (Fig. 1e). The increase in *Fit* expression on tryptone feeding was lower in the DMSO groups compared with those shown in Fig. 1c. We suspected that this is due to less food consumption when DMSO or Rapamycin was added (Supplementary Fig. 2f). Nevertheless, the food consumption was comparable between DMSO and Rapamycin groups, thus the difference in *Fit* expression levels between these two groups is due to the inhibition effects of Rapamycin on the TOR pathway. Taken together, these results indicate that among the three macronutrients, only consumption of protein food promotes *Fit* expression, with this regulation being under the control of the TOR pathway.

### FIT is an adult FB protein involved in feeding control

**Figure 2:**
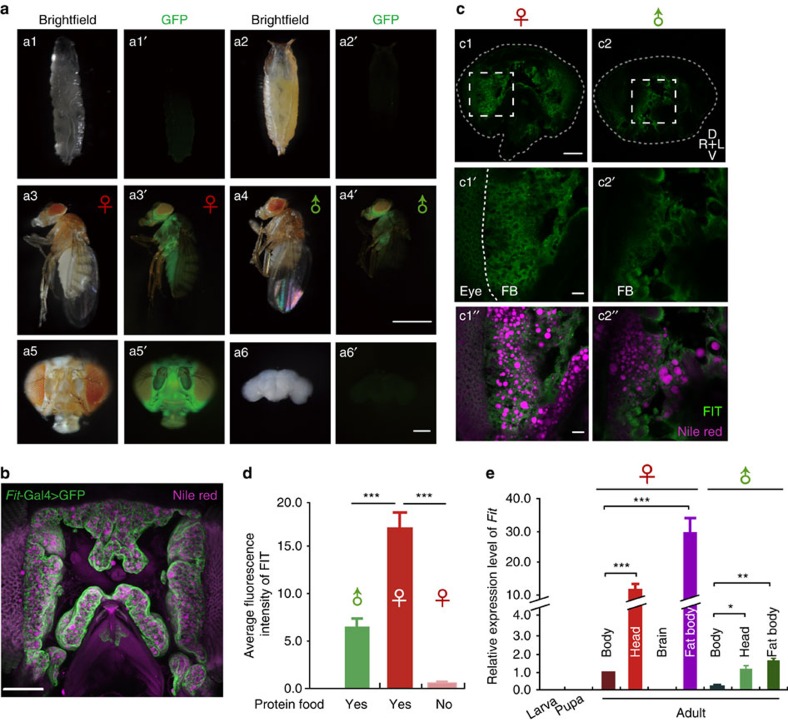
Patterns of *Fit* expression. (**a**) In *Fit-*Gal4>GFP flies, GFP was not detected in larvae (**a**1) and pupae (**a**2), while strong GFP signal was observed in adult flies with obvious difference between females (**a**3) and males (**a**4). In addition, GFP signal was detected at high levels in female head (**a**5) but not in the brain (**a**6). Scale bars, 1 mm in **a**1–**a**4′ and 100 μm in **a**5,**a**6′. (**b**) A representive head section stained with Nile Red. A confocal image stack of ∼80 μm (1 μm per step) was projected in a single image. Scale bars, 100 μm. (**c**) FIT immunostaining detected FIT expression in the head fat body in single confocal images. Dashed lines in **c**1,**c**2 indicate the margin of the head sections. White rectangles in **c**1,**c**2 indicate the areas zoomed in **c**1′,**c**2′, respectively. Scale bars, 100 μm in **c**1,**c**2 and 20 μm in **c**1′–**c**2′′. (**d**) Quantification of fluorescence signal showed that FIT is expressed at high level in female flies fed with protein food. *n*=12–18. One-way ANOVA, LSD's *post hoc* test. (**e**) *Fit* expression levels measured by qPCR at different stages and in different body parts of female and male flies. *n*=3. *P* (Tissue*Sex)=2.98E-8 (two-way ANOVA, Bonferroni test). *P<0.05. **P<0.01. ***P<0.001. NS indicates no statistical significance. The data are mean±s.e.m.

To investigate the expression patterns of *Fit*, we generated a Gal4 reporter fly strain *Fit*-Gal4, which displayed strong expression in adult flies, not however, in larvae and pupae (Fig. 2a, upper and middle panels). Notably, the reporter signal was particularly enriched in the head (Fig. 2a5), but undetectable in the brain (Fig. 2a6). A previous study reported that *Fit* messenger RNA (mRNA) was detected in fat cells within the head^29^. We therefore performed lipid staining in the head sections of *Fit*-Gal4>mGFP flies, where lipid droplets were found in *Fit*-Gal4 expressing cells (Fig. 2b). To determine FIT expression at the protein level, we generated a monoclonal antibody (mAb) and detected endogenous FIT in fat cells within adult heads using immunostaining (Fig. 2c). Consistent with the observation from *Fit*-Gal4 (Fig. 2a, middle panels), we also detected much higher FIT expression in female heads relative to male heads using the antibody (Fig. 2d). In addition, the results from quantitative real-time PCR experiments showed that *Fit* is expressed in adult heads and enriched in the FB with a sexual difference bigger than 10-fold (Fig. 2e). Thus, our results showed that *Fit* is an adult-specific FB gene highly expressed in females, suggesting that it plays a bigger role in female flies.

To determine the physiological function of *Fit*, we generated knock-out (KO) flies by homologous recombination, with two independent lines (*Fit*^*81*^ and *Fit*^*52*^) being screened by PCR (Supplementary Fig. 3a) and validated for *Fit* null mutant alleles by quantitative PCR (data not shown). In addition, immunostaining with the anti-FIT antibody detected negligible signal in the *Fit*^*81*^ mutant flies (Supplementary Fig. 3b). These KO flies developed normally, with body weight as well as protein and lipid contents comparable with wild type (WT) flies (Supplementary Fig. 3c–f). As we showed above, *Fit* displayed a fast response to protein intake (Fig. 1c). Thus we wondered whether *Fit* plays a role in controlling feeding behaviour. Using a CAFE assay, we found that the basal food consumption in *Fit*^*81*^ mutant flies was comparable with WT (Supplementary Fig. 4a). However, after starvation, *Fit* KO flies exhibited higher food intake than *w*^*1118*^ WT flies, and consistent to its high expression in females, this change was more evident in female flies (Supplementary Fig. 4b). Together, these findings suggested that *Fit* is a negative regulator in feeding behaviour.

### *Fit* knock-out flies exhibit deficiencies in PIFI

Given that *Fit* specifically responds to protein-intake, we next asked whether *Fit* exerts a specific role in protein feeding, either for detecting protein in food during feeding initiation, or for controlling total protein consumption during feeding termination. We first tested the protein preference using a two-choice assay, with tryptone mixed with sucrose versus sucrose only. We found that starved WT flies, regardless of sex, preferred tryptone containing food, and moreover, *Fit*^*81*^ mutant flies displayed the same preference pattern as WT flies (Supplementary Fig. 4c), indicating that these mutant flies are normal in detecting protein food and in initiating feeding, with food preference indexes comparable to WT flies.

**Figure 3:**
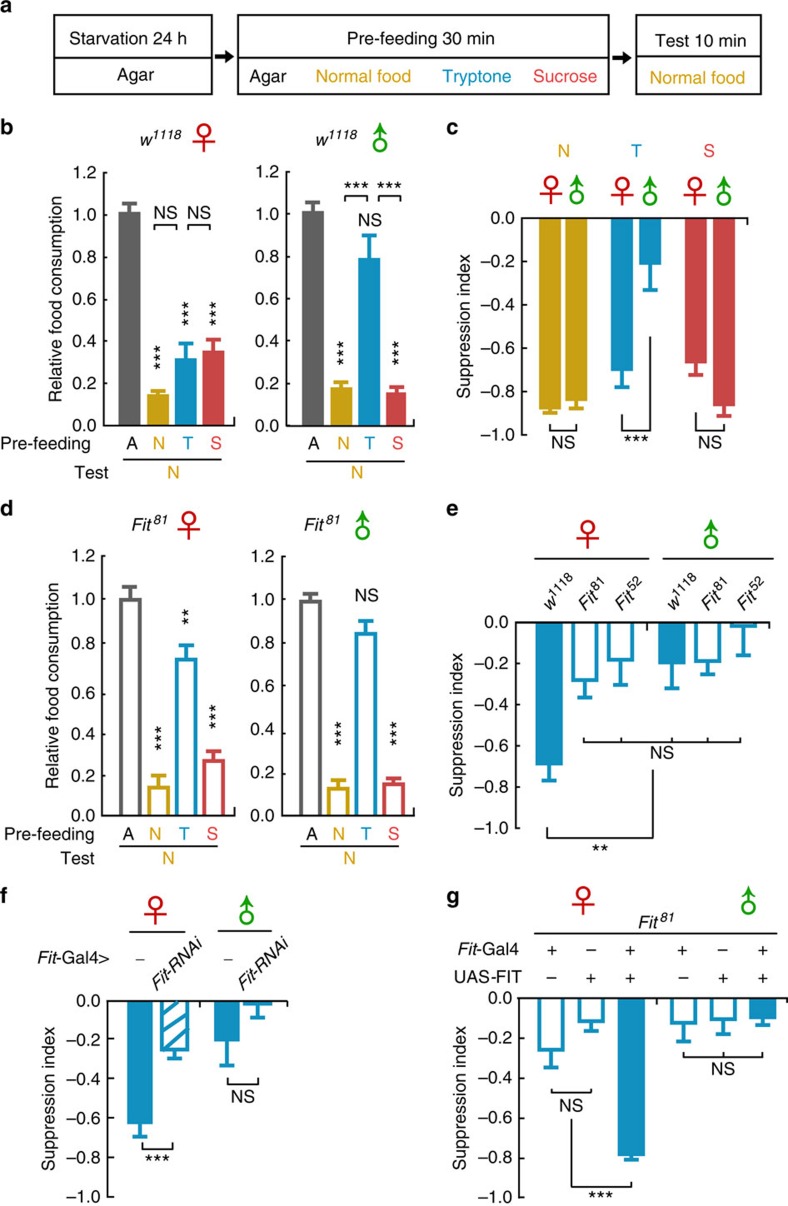
*Fit* KO female flies exhibit deficiencies in protein feeding behaviour. (**a**) Diagram of the pre-feeding paradigm. (**b**) In WT flies, pre-feeding of normal food (N), tryptone (T) or sucrose (S) showed a suppressive effect on subsequent test feeding of normal food when compared with groups pre-fed with agar (A). Thus suppression was observed in both sexes, except that tryptone pre-feeding showed little effect on test feeding in male flies. *n*=8–12. One-way ANOVA, LSD's *post hoc* test. (**c**) SI of tryptone were significantly different between WT female and male flies, while those of normal food or sucrose were comparable between sexes. *n*=8. P (Food*Sex)=8.17E-5 (two-way ANOVA, Bonferroni test). (**d**) Upon tryptone pre-feeding, *Fit*^*81*^ mutant female flies exhibited food intake similar to that in male flies. *n*=8–13. One-way ANOVA, LSD's *post hoc* test. (**e**) SI of tryptone in *Fit* KO females were significantly different from that in WT females, but were comparable to those in WT and KO males. All experiments in **b**–**e** have been performed in parallel. *n*=7–9. One-way ANOVA, LSD's *post hoc* test. (**f**) The suppressive effect of tryptone pre-feeding was significantly reduced in flies with *Fit* knocked down in the FB. *n*=7–9. Data are analysed by unpaired Student's *t*-test. (**g**) In *Fit*^*81*^ mutant background, expressing FIT in the FB rescued the strong suppressive effect of tryptone pre-feeding in female flies. *n*=6–8. One-way ANOVA, LSD's *post hoc* test. *P<0.05; **P<0.01; ***P<0.001. NS indicates no statistical significance. The data are mean±s.e.m.

We then examined whether *Fit* functioned in protein intake-induced feeding inhibition (PIFI). To evaluate the suppression effects of specific food types, we modified the pre-feeding paradigm, as different types of food were used for pre-feeding, individually, while all groups were tested by the normal food (Fig. 3a). In all three WT strains we examined, *w*^*1118*^, CS and *w*CS, pre-feeding with either normal food or sucrose resulted in strong suppressive effects in both female and male flies. Remarkably, pre-feeding of tryptone suppressed food intake greatly in females, but only little in males (Fig. 3b and Supplementary Fig. 5). For quantitative analysis, we calculated the relative difference between the agar group (no pre-feeding control) and all food groups, defined as the suppression index (SI). We found that the SIs of tryptone or AA mix differed significantly between sexes, whereas only slight or no differences were observed in the sucrose or normal food groups (Fig. 3c and Supplementary Fig. 5). Similar results were obtained when the AA mix was used in pre-feeding, or when tryptone-sucrose-mixed food was used instead of normal food in both pre- and test-feeding (Supplementary Fig. 6a,b).

We further examined the feeding suppression effects of different concentrations of tryptone. The data showed that tryptone food suppressed feeding in a dose-dependent manner (Supplementary Fig. 6c), in agreement with the gradually increased *Fit* expression levels along the increase of tryptone concentration (Supplementary Fig. 2d). Moreover, as *Fit* is a sexually dimorphic gene, we wondered whether the mating status of female flies affects its expression and protein-related feeding behaviour. The results showed that *Fit* expression levels are comparable between mated and virgin females, and the PIFI effects were also comparable between these two groups (Supplementary Fig. 6d,e). Therefore, mated females were used in all experiments except this one. Together, these results demonstrated that PIFI manifests itself differently in female and male fruit flies, and that the dimorphic expression of *Fit* might be the cause of this difference.

We therefore examined whether *Fit* was indeed involved in the regulation of PIFI by testing KO flies with the same paradigm. Strikingly, the strong suppression of tryptone pre-feeding was greatly reduced in *Fit*^*81*^ female flies (Fig. 3d), with the SI statistically different from that in WT females, however, undistinguished from WT and *Fit*^*81*^ males (Fig. 3e). In contrast, normal food and sucrose still exerted effective suppression on subsequent feeding in both sexes (Fig. 3d and Supplementary Fig. 7a). Similar results were obtained in *Fit*^*52*^ mutant flies, and also when *Fit* was specifically knocked down in the FB (using *Fit*-Gal4), but not when *Fit*-RNAi was expressed in the nervous system (using either *Elav*-Gal4 or *Dilp2*-Gal4; Fig. 3e,f and Supplementary Fig. 7b–e). Moreover, in *Fit*^*81*^ mutant flies, FB expression of FIT was sufficient to restore the PIFI effect and the sexual difference observed in WT flies (Fig. 3g and Supplementary Fig. 7f). Furthermore, we transiently blocked the up-regulation of *Fit* using the TOR pathway-specific inhibitor Rapamycin, and found that the PIFI effect was greatly reduced (Supplementary Fig. 8a,b). In addition, FB-specific interference of the AA-TOR pathway by knocking-down the AA transporter, *slif*, or overexpressing the suppressor of TOR complex, TSC1/2, led to the same behavioural result (Supplementary Fig. 8c,d). Therefore, these findings indicate that either low levels of *Fit* expression or a deficiency in *Fit* regulation results in weak feeding control, specifically during protein consumption.

### FIT expression levels affect protein feeding

**Figure 4:**
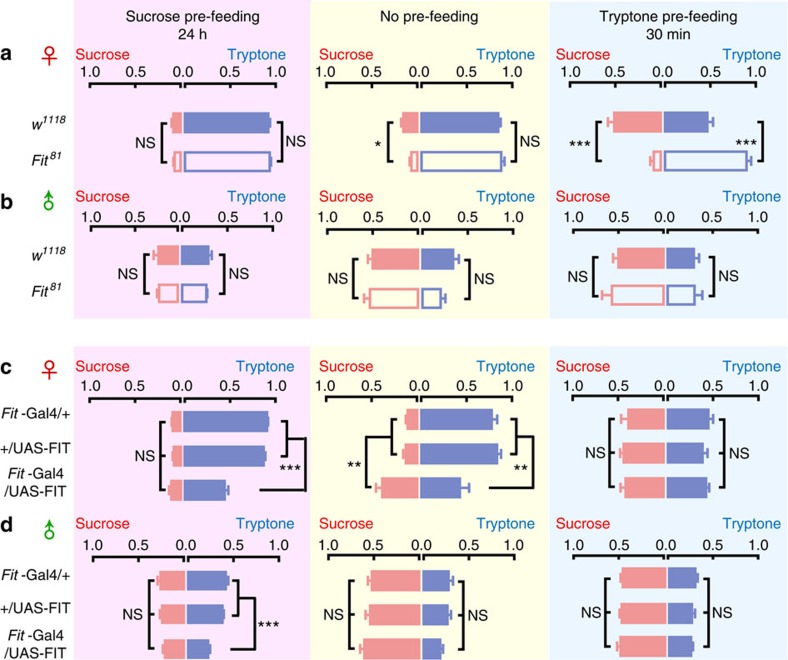
Choice ratio for protein food is increase in *Fit*^*81*^ mutant flies and decreased in FIT-overexpressing flies. (**a**,**b**) After tryptone pre-feeding, the CR for protein and sugar food significantly decreased and increased, respectively, in WT but not *Fit*^*81*^ mutant flies. Flies were kept under normal fed condition (middle row in yellow), and pre-fed with either sucrose (left row in pink) or tryptone (right row in blue). *n*=6–15. Data were analysed by unpaired Student's *t*-test. (**c**,**d**) Overexpression of FIT suppressed protein CR, when compared with their parental control flies. *n*=6. One-way ANOVA, LSD's *post hoc* test. *P<0.05; **P<0.01; ***P<0.001. NS indicates no statistical significance. The data are mean±s.e.m.

We next investigated whether FIT played a role in food preference in non-starved flies using the two-choice assay. Under normal feeding conditions, high ratio of WT female flies displayed tryptone feeding (Fig. 4a, middle panel). This high choice ratio (CR) significantly decreased after tryptone pre-feeding (*P*<0.001), while the CR for sucrose feeding increased (*P*<0.01); on the other hand, the CR changed a little following sucrose pre-feeding (*P*<0.05 for both; Fig. 4a). Under both no pre-feeding and sucrose pre-feeding conditions, *Fit*^*81*^ female flies displayed similar feeding choices to WT flies, suggesting that the protein requirement is normal in these mutant flies. However, the CR for protein food sustained at high level after tryptone pre-feeding, in other words, these KO flies failed in adjusting their feeding choice in accordance with their high protein state (Fig. 4a). Consistent with the findings obtained from starved flies (Fig. 3e), feeding behaviour of *Fit*^*81*^ male flies were comparable to that of WT male flies under these non-starved conditions, with little feeding choice for protein food relative to female flies (Fig. 4b). Notably, WT male flies showed reduced feeding of sugar food after sucrose pre-feeding; however, there was no compensatory increase in protein intake, neither in WT nor in KO flies (Fig. 4b, left panel). Together, these results demonstrated that female flies have high protein requirement and feeding CR, and FIT is required for preventing them from protein overconsumption; in addition, the protein requirement is low in males even after protein deprivation for one day, and low levels of FIT do not promote protein feeding on their own.

Next we examined whether high levels of FIT were sufficient to suppress feeding, and whether it was in a protein-specific manner. Under normal conditions, female flies with FIT overexpression exhibited a significant decrease in the CR for protein, and a similar increase in that for sugar, when compared to their parental controls (Fig. 4c, middle panel). After sucrose pre-feeding, protein CR was also reduced in these flies, however, sugar CR remained unchanged (Fig. 4c, left panel). Furthermore, after tryptone pre-feeding, flies with high FIT expression showed protein and sugar intake comparable to controls (Fig. 4c, right panel). In males, overexpressing FIT also resulted in suppression of protein CR, which was evident only after sucrose pre-feeding. Under the other two conditions, male flies displayed low protein CR in control groups, with FIT overexpression not exerting any effect (Fig. 4d, middle and right). Taken together, our data indicate that FIT plays an essential role in feeding inhibition, and acts selectively on protein intake, with this function manifesting itself in both female and male flies, albeit to different extents.

### Secreted peptide FIT inhibits feeding via insulin signalling

**Figure 5:**
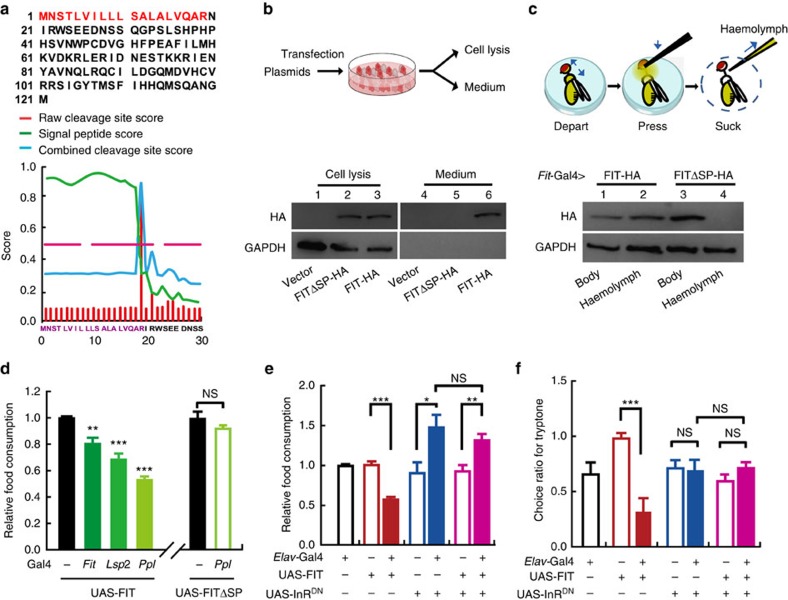
FIT is a secreted peptide and exerts its feeding suppression function by interacting with the insulin signalling in the nervous system. (**a**) The first 19 AAs (red) of FIT were predicted to be a SP according to the three output scores produced by the neural networks software in SignalP 4.1. (**b**,**c**) HA signal was detected in conditioned medium (**b**) and fly haemolymph (**c**) of FIT-HA groups, but not FITΔSP-HA control groups. As control, HA signal was detected in cell lysis (**b**) and whole-body lysis (**c**) of both expression groups. The western blot experiments were replicated three times. (**d**) FB-expression of FIT, but not FITΔSP, suppressed feeding behaviour. *n*=8–16. (**e**) Flies co-expressing FIT and InR^DN^ in the nervous system exhibited over-feeding behaviour similar to InR^DN^-overexpressing flies. *n*=6–8. (**f**) Overexpression of FIT significantly suppressed the CR for tryptone, while this effect was abolished when InR^DN^ was co-expressed with FIT. Tryptone and sucrose were used in the two-choice assay. *n*=5–12. Data are analysed by unpaired Student's *t*-test. *P<0.05; **P<0.01; ***P<0.001. NS indicates no statistical significance. The data are mean±s.e.m.

Given that FIT is a FB-expressing protein that lacks expression in the brain (Fig. 2), the question arose as to how FIT exerts its function in regulating behaviour. We analysed the protein sequence of FIT, which contains 121 AAs, and found that the first 19 AA is predicted to be a signal peptide (SP) for secretion (Fig. 5a), implying that FIT is a secreted protein. To test this possibility, we utilized a cell culture system to express HA-tagged proteins *in vitro* and examined the conditioned medium using western blot. We detected a strong HA signal in the FIT conditioned medium, whereas no signal was found in the control groups, with either empty vector or FIT lacking the SP (FITΔSP; Fig. 5b and Supplementary Fig. 9a). To verify that FIT was secreted into fly haemolymph, we expressed HA-tagged proteins in the FB and examined the haemolymph for the presence of FIT protein. In flies expressing full-length FIT-HA, HA signal was detected in the fly haemolymph. In contrast, in flies expressing the truncated version FITΔSP-HA, the HA signal was only detected in whole-body lysates, not however, in the haemolymph (Fig. 5c and Supplementary Fig. 9b). These results provide strong evidence that FIT is a secreted peptide, and that the first 19 AAs are essential for its secretion.

We then tested whether the secretion of FIT was required for its functions on feeding suppression. After starvation for 24 h, flies were examined in normal food. Our results showed that overexpressing FIT in the FB, using three FB-Gal4 strains individually, resulted in a significant reduction in food intake, whereas overexpression of FITΔSP lacked such impact (Fig. 5d). Similarly, ectopic expression of FIT with the pan-neuronal *Elav*-Gal4 also induced significant feeding suppression, while again FITΔSP displayed no such function in feeding regulation (Supplementary Fig. 9c). We then used *Elav*-GSG, an inducible Gal4, to achieve neural expression of FIT specifically in the adult stage, which also resulted in effective suppression on food intake (Supplementary Fig. 9d). Together, these results demonstrate that FIT is a secreted peptide existing in the fly haemolymph, and that only the secreted form of FIT is functional in feeding control.

It has been proposed that FB-derived nutritional signals exert their functions by regulating insulin signalling in the brain^16^,^17^. As shown in Supplementary Fig. 9c, ectopic expression of FIT in IPCs using *Dilp2*-Gal4 resulted in significant feeding suppression, suggesting that FB-derived FIT peptide might exert its function on feeding control by targeting this Gal4-labelled brain region. To test whether FIT regulates feeding behaviour through insulin signalling, we then performed a genetic interaction experiment by co-expressing FIT and InR^DN^, the dominant negative (DN) form of the InR, using the *Elav*-Gal4. Expression of InR^DN^ leads to a downregulation of insulin signalling, and as expected, pan-neuronal expression resulted in a significant increase in feeding. Notably, when InR^DN^ and FIT were co-expressed, we observed an increase similar to that observed when overexpressing InR^DN^ alone, although FIT overexpression strongly suppressed feeding on its own (Fig. 5e). Moreover, we performed the genetic interaction experiment using the two-choice assay, under the same condition shown in the left panel of Fig. 4c. Consistently, FIT overexpression significantly suppressed tryptone intake; moreover, this effect was abolished when InR^DN^ was also overexpressed (Fig. 5f and Supplementary Fig. 9e). Together, these results indicate that in the absence of CNS insulin signalling, high levels of FIT expression no longer suppress protein feeding.

### FIT mediates DILP2 release induced by protein consumption

**Figure 6:**
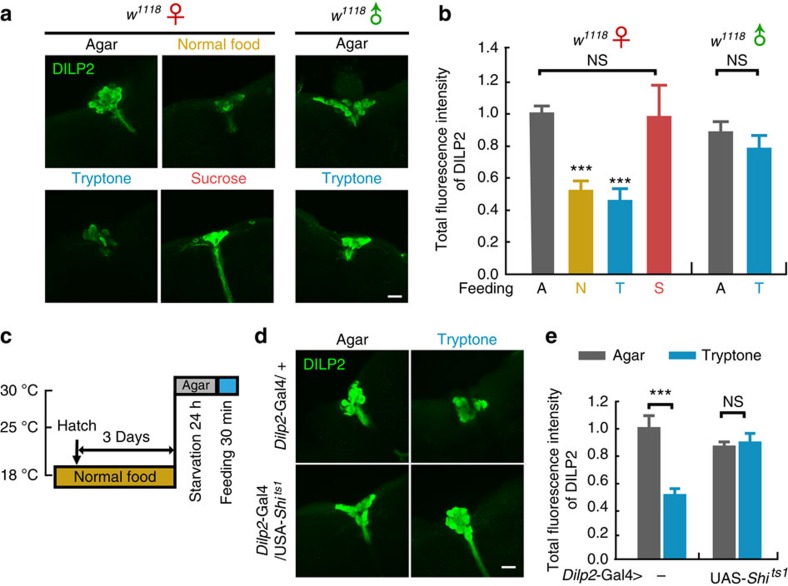
Protein intake induces significant DILP2 release in IPCs. (**a**,**b**) Tryptone feeding resulted in a significant decrease of DILP2 signals in female but not male flies. Representive images of DILP2 immunostaining in IPCs after 30 min feeding of different types of food and its quantification data are shown. *n*=7–49 for female groups, *n*=18–23 for male groups. Scale bar, 20 μm. *P* (Food*Sex)=4.40E-3 (two-way ANOVA, Bonferroni test). (**c**) Diagram of temperature induction for blocking the secretion of IPCs using *Dilp2*-Gal4>UAS-*Shi*^*ts1*^ flies. (**d**,**e**) Tryptone feeding induced a significant reduction in DILP2 staining signals, which was absent in flies with deficient secretion in IPCs. *n*=12–16. Scale bar, 20 μm. P (Food*Genotype)=1.23E-3 (two-way ANOVA, Bonferroni test). ***P<0.001. NS indicates no statistical significance. The data are mean±s.e.m.

Within the *Drosophila* DILP family, DILP2 is expressed at the most abundant levels^30^, and DILP2 release has been found previously to be regulated by nutrition signals from the FB (refs 16, 17, 18, 19). To check whether protein consumption induces DILP2 release, and also whether this process was mediated by FIT, we utilized the same treatments used in the behavioural pre-feeding assay, and examined DILP2 levels in IPCs by immunofluorescence^16^. In female flies, the DILP2 signal was significantly reduced in groups with 30 min feeding of normal food or tryptone, not however, in sucrose groups (Fig. 6a,b and Supplementary Fig. 10a). This result is in agreement with previous findings in adult flies^31^ and larvae^16^,^32^. In contrast, DILP2 levels remained unchanged following tryptone feeding in male flies (Fig. 6a,b), which was consistent with our behavioural results. To test whether the reduction of DILP2 signal in the IPCs was due to increased cell secretion, we utilized a mutant allele of *Shibire* (*Shi*^*ts1*^). *Shi*^*ts1*^ encodes a temperature sensitive, DN form of the dynamin protein, and is known to be able to block synaptic vesicle release^33^. When *Shi*^*ts1*^ was expressed in IPCs to temporarily block their secretion at the temperature of 30 °C, tryptone feeding-induced reduction in DILP2 levels was abolished (Fig. 6c–e and Supplementary Fig. 10b), suggesting that the observed reduction in DILP2 levels was a result of increased DILP2 release. Thus, these results reveal that in WT flies, protein intake triggers DILP2 release in a sexually dimorphic manner, which strongly suggested that FIT played a role in this regulation.

**Figure 7:**
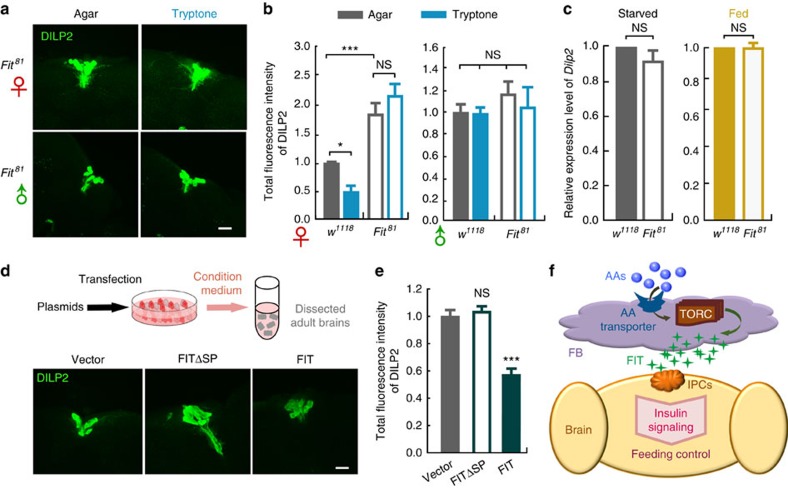
Secreted FIT peptide is responsible and sufficient to induce DILP2 release. (**a**,**b**) DILP2 signal in *Fit*^*81*^ female flies did not decrease after 30 min feeding of tryptone. *n*=20–42 for female groups, *n*=19–33 for male groups. P(Food*Genotype)=0.0177 in female, P(Food*Genotype)=0.6906 in male (two-way ANOVA, Bonferroni test). Scale bar, 20 μm. (**c**) The mRNA levels of *Dilp2* in fly heads were comparable in *Fit*^*81*^ and WT female flies. Data were analysed by unpaired Student's *t*-test. *n*=3. (**d**,**e**) DILP2 fluorescent signal in brains incubated with FIT conditioned medium was significantly lower than that with either the empty vector or FITΔSP. *n*=22–47. One-way ANOVA, LSD's *post hoc* test. Scale bar, 20 μm. (**f**) Diagram of FIT function across two organs. In response to increased AA levels, FIT is expressed and secreted from the FB, and regulates feeding behaviour via CNS insulin signalling. *P<0.05; ***P<0.001. NS indicates no statistical significance. The data are mean±s.e.m.

We then tested whether FIT mediates DILP2 release upon protein intake in female flies. We found that after tryptone feeding, DILP2 levels were not reduced in *Fit*^*81*^ female flies (Fig. 7a,b and Supplementary Fig. 10c), which is in agreement with their behavioural defect in PIFI (Fig. 3d). Moreover, we observed a significantly stronger DILP2 signal within the IPCs in KO females than in WT females (Fig. 7b and Supplementary Fig. 10c). In contrast, *Dilp2* mRNA levels were comparable between KO and WT, independent of whether the flies had been starved or fed before analysis (Fig. 7c). These results suggest that DILP2 release is deficient in *Fit*^*81*^ mutant flies, resulting in an accumulation of DILP2 in the IPCs.

To test whether FIT was sufficient to induce DILPs release, we used FIT conditioned medium to treat dissected brains and examined the protein levels of DILP2 by staining (Fig. 7d). We found that after an incubation for 0.5 h, DILP2 levels were significantly lower in brains treated with FIT-conditioned medium, compared with the control groups with either empty vector or FITΔSP (Fig. 7e and Supplementary Fig. 10d). To examine whether this change was due to an increased release of DILP2, we utilized the secretion-deficient fly strain *Dilp2*-Gal4>UAS-*Shi*^*ts1*^ (Supplementary Fig. 10e). We found that DILP2 signal in such flies did not change following incubation with FIT medium, while the same treatment induced a significant reduction of DILP2 signal in the parental control group (Supplementary Fig. 10f), indicating that the reduction in DILP2 signal on FIT incubation was due to the increased release of DILP2. Taken together, on the basis of our findings, we propose a model that upon protein intake, FIT expression in the FB is elevated, and secreted FIT peptide promotes the release of DILP2, thereby suppressing protein intake through insulin signalling (Fig. 7f).

## Discussion

Protein is recognized as the most satiating macronutrient, however, the relationship between protein intake and feeding control remains ill-defined^6^,^11^. Protein consumption induces various satiety signals, which are derived either from taste, the food-induced stretching of the stomach, or the nutritional value of food taken in refs 1, 4. These signals have comprehensive and redundant physiological functions, therefore challenging the dissection of the complex regulation of feeding behaviour. Here, We identified FIT as a satiety signal representing the internal nutrition levels of protein, allowing the manipulation of this signal without interfering with other feeding-induced satiety signals. In *Drosophila*, sugar-sensing pathways and their roles in feeding behaviour have been extensively studied^2^,^3^. Therefore, identification of AA sensing pathways provides an opportunity to study the integration of different nutrient signals and the principles of neural regulation in feeding decision.

Our results show that when protein and sugar foods are supplied separately, the protein satiety signal selectively suppresses protein intake, though it has no suppression effect on sugar intake. When the two nutrients are provided in a mixture, the feeding decision is made by comparing the strength of appetite and satiety signals for these two nutrients. If the appetite signal of one nutrient (for example, sugar) is strong, it may overcome the satiety signal of another nutrient (for example, protein), resulting in overconsumption of the latter. Thus, our results provide strong experimental evidence and a mechanistic explanation for the higher health risk of consuming mixed-nutrient food, such as certain types of processed food, where nutrients may not be properly balanced^7^,^34^.

FIT was originally found and named as a female-specific gene^29^. Our data presented here show that FIT is expressed in both sexes, however, at much higher levels in females than males. In agreement with this finding, our behavioural results demonstrate that FIT also exerts its feeding suppression function in male flies. We suspect that this sexual dimorphism is possibly due to the big difference of protein requirement between males and females, as one female fly requires large amounts of protein to produce hundreds of embryos. This is in agreement with previous reports, showing that adult female flies are more sensitive to protein deprivation and switch their food preference to protein food much faster than males^14^. Such sexual difference in protein requirement exists in adults, but not during developmental stages, and accordingly, *Fit* is expressed only in adult flies. These findings indicate that throughout evolution, the regulatory mechanism for matching protein intake with protein requirement has been imprinted into the genome, allowing its precise manifestation in terms of both sex difference and specific developmental stage.

Sexual dimorphism in feeding has been observed in many species, for example, in mice, which display different food anticipatory behaviour between sexes^35^. Multiple peptides, like Leptin, Ghrelin, Cholecystokinin, and Glucagon-like peptide-1, are also found to be of different concentrations in blood between female and male mouse^35^,^36^. Nevertheless, protein-specific satiety peptides have not been identified in vertebrates. Similar to FIT, Leptin is an adipose tissue-derived peptide with important functions in regulating feeding behaviour. Expressing human Leptin in flies rescued the developmental defects in *Upd2* mutant^17^; however, it did not rescue the behavioural defects in *Fit* mutant flies (data not shown). In *Drosophila*, Upd2 is a FB-secreted peptide mediating the nutrition signal of sugar and lipids^17^. Therefore, we suspect that Leptin may have a conserved role of Upd2 in nutrition sensing, while other peptides may serve as the functional homologue of FIT for AA sensing and feeding control in mammals. Two recently reported AA-responding peptides, GBPs and Sun, are expressed in *Drosophila* larval FB and are essential for the control of nutrient-directed growth^18^,^19^. However, flies display extensive feeding at the larval stage, thus nutrient signals mediated by these two peptides are not suspected to elicit negative feedback on feeding behaviour. Interestingly, larval FB disappears during metamorphosis^37^,^38^, and in adult flies, the FB reappears with different origin. Thus, to accomplish new metabolic requirements in adults, the remodelled FB may employ a different set of molecules, for example, the satiety signal for achieving stronger feeding inhibition following protein-intake. Further investigation on the regulatory mechanisms of *Fit* expression and the downstream signals in the brain should improve our understanding of the sexual dimorphism in feeding behaviour, as well as in nutrition sensing-related physiological processes, such as aging, in animals from insects to human beings.

The CNS is ultimately responsible for the final evaluation of nutrition state and for controlling feeding behaviour. In both mammals and insects, insulin signalling has also been shown to play essential roles in feeding control^21^,^22^,^25^,^26^. Nevertheless, its roles in nutrient-specific regulation of food intake have not been reported. In mammals, a high protein diet induces stronger activation than normal food in the nucleus tractus solitaries and in the arcuate nucleus^11^. A recent study in adult flies reported that upon AA consumption, neurons in the PI region were activated with a significant reduction in DILP2 signal, whereas sugar consumption had no such effect^31^. Our results showed that DILP2 is secreted in response to protein consumption but not sugar intake, in agreement with two independent studies in larvae^16^,^32^. These observations hint at the existence of protein-specific signals targeting the CNS, and *Drosophila* DILP2 signalling may serve as a protein-specific signal in the brain.

*Drosophila* IPCs in the PI brain region also produce another two DILPs, DILP3 and DILP5. Interestingly, a study in *Drosophila* larva reported that DILP2 and DILP3 are localized in separate vesicles within IPCs; in addition, DILP2 secretion is induced on protein intake but not sugar feeding, whereas DILP3 secretion responds to these two nutrients oppositely^32^. There are a number of regulators functioning in the PI brain region^39^, including various neuromodulators, such as dopamine, GABA, serotonin and octopamine, as well as multiple neuropeptides, such as Drosulfakinin^40^, allatostatin A^41^ and short Neuropeptide F^42^. Therefore, potential distinct IPCs or their targeting neurons combined with different DILPs, along with distinct local regulatory neural modulators and networks, are capable to control feeding behaviour in a nutrient-specific manner. To uncover the downstream signals of FIT in the brain, more specifically in the PI region, it is critical to identify its receptor. A recent study undertaken in *Drosophila* larvae reported that Methuselah (Mth) is expressed in the IPCs and serves as the receptor of the FB-secreted peptide Sun. Together, they modulate DILP release and systemic growth in response to high nutrient levels^19^. Mth is a member of the secretin-incretin receptor subfamily, belonging to the G protein-coupled receptor family. In our previous behavioural screen for G protein-coupled receptors required for PIFI in adult flies, one of the chosen candidates belongs to the same subfamily^43^. The identification of the FIT receptor, together with physiological studies of FIT targeting neurons in the PI brain region will enable us to better understand how AA state signals are coded, processed and integrated with signals from other nutrients, and how these nutrition signals ultimately contribute to an appropriate feeding decision, both in lower and higher organisms.

## Methods

### Foods and fly strains

Flies were reared on normal food with the recipe of Bloomington *Drosophila* Stock Center at 25 °C, 60% humidity, and 12/12 light/dark cycle. Restrictive foods were prepared in 1% agar: single-nutrient foods consisted of 1.7% tryptone, 10% sucrose or 1.7% soy lipid^16^; tryptone-sucrose-mixed food contained 1.7% tryptone and 10% sucrose. For the dose-dependent experiments, 0% (agar only), 0.5, 1.1, 1.7 and 2.3% tryptone in 1% agar were used. AA mix was a mixture of all known AAs at the concentration present in tryptone (Supplementary Table 1). Adult flies were collected at hatching, and mated female and male flies were used in all experiments at age of 3–5 days, except virgin females of 3–5 days were also examined as presented in Supplementary Fig. 6d,e.

Three WT fly strains *w*^*1118*^, Canton S (CS) and *w*CS were used in this study. Fly strain *Dilp2*-Gal4 was obtained from Dr Rajan A. and Dr Perrimon N., UAS-TSC1/2 and UAS-*Slif*-anti from Dr Leopold P., *Ppl*-GAL4 from Dr Huang X., UAS-InR^DN^ from Dr Shen P., 3.1-*Lsp2*-GAL4 from Dr Dauwalder B., UAS-*Shi*^*ts1*^ from Dr Tully T., and *Elav*-GSG (Gene Switch) from Dr Davis R.L.. Fly strains *Elav*-GAL4 (458) and UAS-mCD8::GFP (5137) were obtained from Bloomington *Drosophila* Stock Center. UAS-*Fit*-RNAi (14434) was obtained from Vienna *Drosophila* Research Center.

*Fit* KO flies were generated by homologous recombination according to the method developed by Dr Yang Hong^44^. In brief, transgenic flies carrying targeting construct were crossed to hs-FLP,hs-SceI (BL-6934), and the progenies with red-eye were screened by PCR (Supplementary Fig. 3a). Two independent KO lines were validated by qPCR and backcrossed to *w*^*1118*^ for five generations.

*Fit*-Gal4 was generated by cloning 1.1 Kb promoter region upstream of *Fit* into the vector PPTGal. UAS-FIT and UAS-FITΔSP fly strains were generated by cloning full length and truncated (the first 4–57 nt was removed) *Fit* cDNAs into the pUAST vector, respectively, with the HA sequence attached.

Primers used for cloning are listed in the Supplementary Table 2. Microinjection was performed by Rainbow Transgenic Flies Inc., USA using flies of the laboratory WT strain *w*^*1118*^.

### Feeding behaviour assays

Adult flies were starved on 1% agar for 24 h in groups of ∼100 per bottle, and then transferred to bottles with test food containing 0.5% Brilliant Blue (Care, Chemodist Industris) for 10 min. After a quick freeze, crops of randomly picked flies were dissected in PBS (pH 7.2) and categorized into female and male groups. Crops of 20 per group were homogenized and centrifuged (13,000*g*) for 5 min, and the supernatants were transferred to a new tube and diluted with PBS to a total of 1 ml. The absorbance was measured at 620 nm with Multilabel Detection Platform (Hidex Chameleon Plate).

In the Pre-feeding paradigm (Fig. 1a), relative Food Consumption (FC) in the test phase was calculated in each group divided by the mean of those in agar control groups. In each parallel experiment, the difference of FC between food group and agar group was calculated and normalized to the FC_agar_, and this difference was then defined as the SI. All experiments were carried out more than six times for each group to get credible results^45^.

A two-choice feeding assay was adapted and modified from the classic two-choice feeding assay^14^ and described in our previous report^43^. In brief, 30 flies were allowed to choose between two types of coloured foods for 30 min in the darkness, and then were sorted according to the colour in their abdomens. The numbers of flies with blue, red or purple abdomens were counted as N_blue_, N_red_ or N_both_. Total fly number including N_blue_, N_red_, N_both_ and those with white abdomens was counted as N_total_. The CR was calculated as CR_blue /red_=(N_blue/red_+0.5 × N_both_)/N_total_. Foods of tryptone and sucrose were mixed with blue and red dyes, respectively, and the combination switched in parallel experiments. The final CR for tryptone or sucrose was calculated as (CR_blue_+CR_red_)/2 with data obtained from a set of parallel experiments. More than six repeats were performed in every experimental group^46^.

Animals were allocated randomly in all experiments. We conducted double-blind experiments in all behavioural experiments.

### Generation of anti-FIT mAb and staining

A mAb against the FIT protein was successfully generated using Display approach. Briefly, *Fit* cDNA without the sequence for the SP was cloned into the vector of pDisplay (Thermo Fisher) with fusion HA tag. Female BALB/c mice were immunized once a month with mouse L cells transfected with pDisplay-FIT-HA. After the fourth immunization, the spleen cells were isolated and fused with myeloma cells. The supernatants of hybridomas culture were collected for screening using immunostaining approach in Hela cells transfected with pDisplay-FIT-HA plasmid. Selected hybridoma clones were subcloned for three rounds, and the stable ones were injected into immunodeficient mice. Ascites fluid was collected two weeks post injection, and mAb were purified using NAb Protein G Spin Columns (Pierce).

For immunostaining experiments, fly heads were separated from the body, and the heads were then quickly embedded and frozen in one drop of O.C.T compound (Sakura). The cryo-section was obtained at −20 °C using a Cryotome E (Thermo). Head slices were fixed in 4% paraformaldehyde for 30 min, and stained with Nile Red (1 μg ml^−1^ in PBS, Sigma) and anti-FIT mAb (1:1,000), followed by anti-mouse Alexa 488 (1:500, Invitrogen). In protein food induction groups, yeast as a protein source was added 20 h before the start of the experiments. For quantification, 3 heads were sliced in each group, and a single confocal image (Leica, SPE) was obtained from each slice. Three randomly selected areas (50 × 50 μm^2^) within the FB region in each image were subjected to measurement of the fluorescent signal using ImageJ.

DILP2 level was determined by immunostaining according to standard whole fly brain staining protocol^16^. In brief, brains of 4-day-old flies were dissected, fixed, blocked and stained with rat anti-DILP2 (1:500, kindly provided by Dr Pierre Leopold)^16^ as the primary Ab and anti-rat Alexa 488 (1:200, Cell Signaling Technology) as the secondary Ab. Samples were mounted in Vectashield mounting medium (Vector Laboratories, Inc.; Peterborough, UK). Confocal (Leica, SPE) Z series of the IPCs were acquired using identical laser power and scan settings at the step of 1 μm. To evaluate DILP2 signal across the 3D structure of IPCs, total fluorescence intensity, overall mean intensity, and total volume of DILP2 signal were measured using ImageJ in combination with a plugin (Measure Stack) developed by Dr Bob Dougherty (OptiNav, Inc.).

### Cell culture and fly haemolymph

FIT-HA and FITΔSP-HA were cloned into the vector pcDNA3.1, and transient transfections were performed in HEK 293 cells (gifted from Dr Liguo Zhang) using Lipofectamine 2000 (Invitrogen). Mycoplasma contamination was tested using Lookout Mycoplasma Detection Kit (Sigma, MP0035), and no contamination was detected. After 24 h, conditioned medium was harvested, and cells were lysed with 1 ml lysis buffer (Merck). Brain incubation was performed according to a modified *ex vivo* organ culture method^16^. Brains of 4-days female files were dissected in ice-cold PBS and then incubated with 200 μl conditioned medium at 25 °C for 30 min, followed by DILP2 staining.

Adult fly haemolymph was prepared according to a modified version of a previously published protocol^47^ (Fig. 5c). In brief, five flies were put into a drop of 6 μl ice-cold lysis buffer (Novagen, 71009), and the fly heads and bodies were gently pulled in opposite directions without separation or disruption of any tissues. The forceps was used to gently push the head and body, and the haemolymph diffused in the lysis buffer was collected. Collections from 80 flies were then combined to a total of 40 μl. For total body protein, ∼20-whole flies were lysed in each group.

For western blot analysis, equal quality of protein samples of conditioned medium, cell lysis, fly haemolymph, and fly lysis were used. Antibodies used were HA antibody (CWBIO, CW0260, 1:2000) followed by Goat Anti-Mouse IgG, HRP (CW0102, 1:5,000), and GAPDH antibody (CW0266, 1:2000) followed by Goat Anti-Mouse IgG, HRP (CW0102, 1:5000). Full size gel images of those shown in Fig. 5b,c are presented in Supplementary Fig. 9a,b.

### Treatment and induction

Rapamycin (Gene Operation, IPA1021-0100MG) was dissolved in DMSO (Sigma, D2650) at 200 mM. This solution was added to agar and tryptone food at a final concentration of 1 mM. Equal volume of DMSO was added to food in control groups. In related experiments, these mixtures were used from the start of the starvation procedure. For RU486-induced Gal4 expression^48^, flies were collected after eclosion and reared for 3–4 days in normal food containing 500 mM RU486 (Sigma, 84371-65-3).

For temperature induction experiments (Fig. 6c and Supplementary Fig. 10e), *Dilp2*-Gal4>UAS-*Shi*^*ts1*^ flies were crossed and reared at 18 °C. Three days after eclosion, flies were transferred to and maintained at 30 °C in the subsequent feeding or brain incubation experiments.

### Quantitative real-time PCR

Total RNA was extracted from 8 fly bodies or ∼100 fly heads with TRIzol (Invitrogen). RNA samples were treated with RQ1 DNase (Promega) and reverse-transcribed using PrimeScript RT Master Mix (TaKaRa). Relative quantification PCR was carried out using a SYBR Premix Ex TaqTM II kit (Takara) and an ABI PRISM 7300 real-time PCR Detection system (Applied Biosystems). Relative mRNA levels were calculated using the comparative CT method. *Rp49*, *Dp* and *Actin 5C*, were used as the internal control, and gene expression levels were normalized to treatment control or genetic control. Three separate samples were collected from each condition, and measurements were conducted in triplicates.

### Measurement of triglyceride and protein contents

Triglyceride of flies was measured as reported previously. In brief, adult flies were homogenized and incubated at 70 °C for 5 min. After centrifugation, supernatants were incubated with Triglyceride Reagent (Sigma, T2449) for 30 min at 37 °C, then mixed with Free Glycerol Reagent (Sigma, F6428). Protein contents in flies were detected using a Pierce bicinchoninic acid (BCA) protein assay (Sangon Biotech, SK3051). Adult flies were homogenized in RAPA lysis buffer. After centrifugation, supernatant was added to BCA work solution. The lipid and protein contents were quantified by measuring the absorbance at 540 nm using a spectrophotometer (Hidex Chameleon Plate), and the results were normalized to the weight of the flies.

### Statistical analysis

All experiments were performed with experimental and control groups in parallel. Data were analysed with SPSS (SPSS Inc.) and Matlab (MathWorks Inc.). One-way ANOVA analysis of variance with Fisher's least significant difference (LSD) *post hoc* test, two-way ANOVA analysis of variance with Bonferroni test, or unpaired Student's *t*-tests were used according to the number of conditions and groups. The statistic methods, along with genotype, sex, experimental assays, and antibodies, are listed in the Supplementary Table 3. In all figures, *n* indicates number of independent experiments, and histograms present mean±s.e.m. *P<0.05; **P<0.01; ***P<0.001. NS indicates no statistical significance.

### Data availability

The data that support the findings of this study are available from the corresponding author upon reasonable request.

## Additional information

**How to cite this article:** Sun, J. *et al*. *Drosophila* FIT is a protein-specific satiety hormone essential for feeding control. *Nat. Commun.* **8,** 14161 doi: 10.1038/ncomms14161 (2017).

**Publisher's note:** Springer Nature remains neutral with regard to jurisdictional claims in published maps and institutional affiliations.

## Supplementary Material

Supplementary Information Supplementary Figures and Supplementary Tables
