# Supplementary material for: Drosophila FIT is a protein-specific satiety hormone essential for feeding control
Source: Nat Commun. 2017 Jan 19;8:14161. doi: 10.1038/ncomms14161 (PMC5253699; doi:10.1038/ncomms14161)
Supplement: Supplementary Information — Supplementary Figures and Supplementary Tables [file ncomms14161-s1.pdf]

# Supplementary Information

## Supplementary Figures

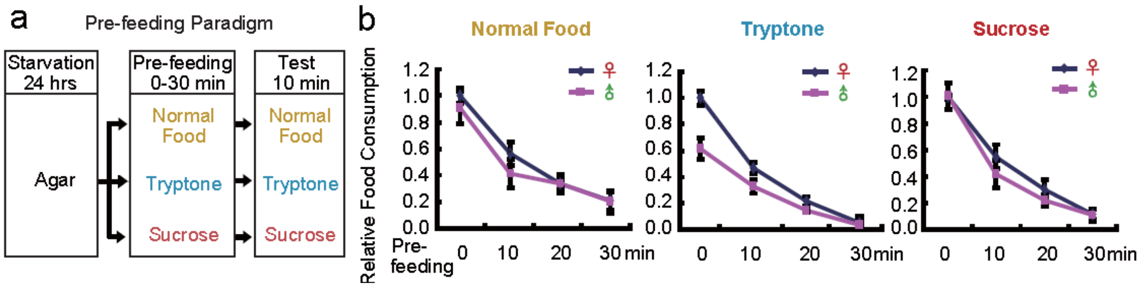


**Supplementary Figure 1 | All three types of foods suppress subsequent feeding in both sexes when the same food is used in the pre-feeding and test feeding.**

(a) Adjusted pre-feeding paradigm. In each group, the same food was used in the pre-feeding and test feeding.

(b) The amount of food consumed decreased with feeding time. After 30 min feeding of normal food, tryptone, or sucrose, both females and males were satiated and stopped eating. n=5-7.

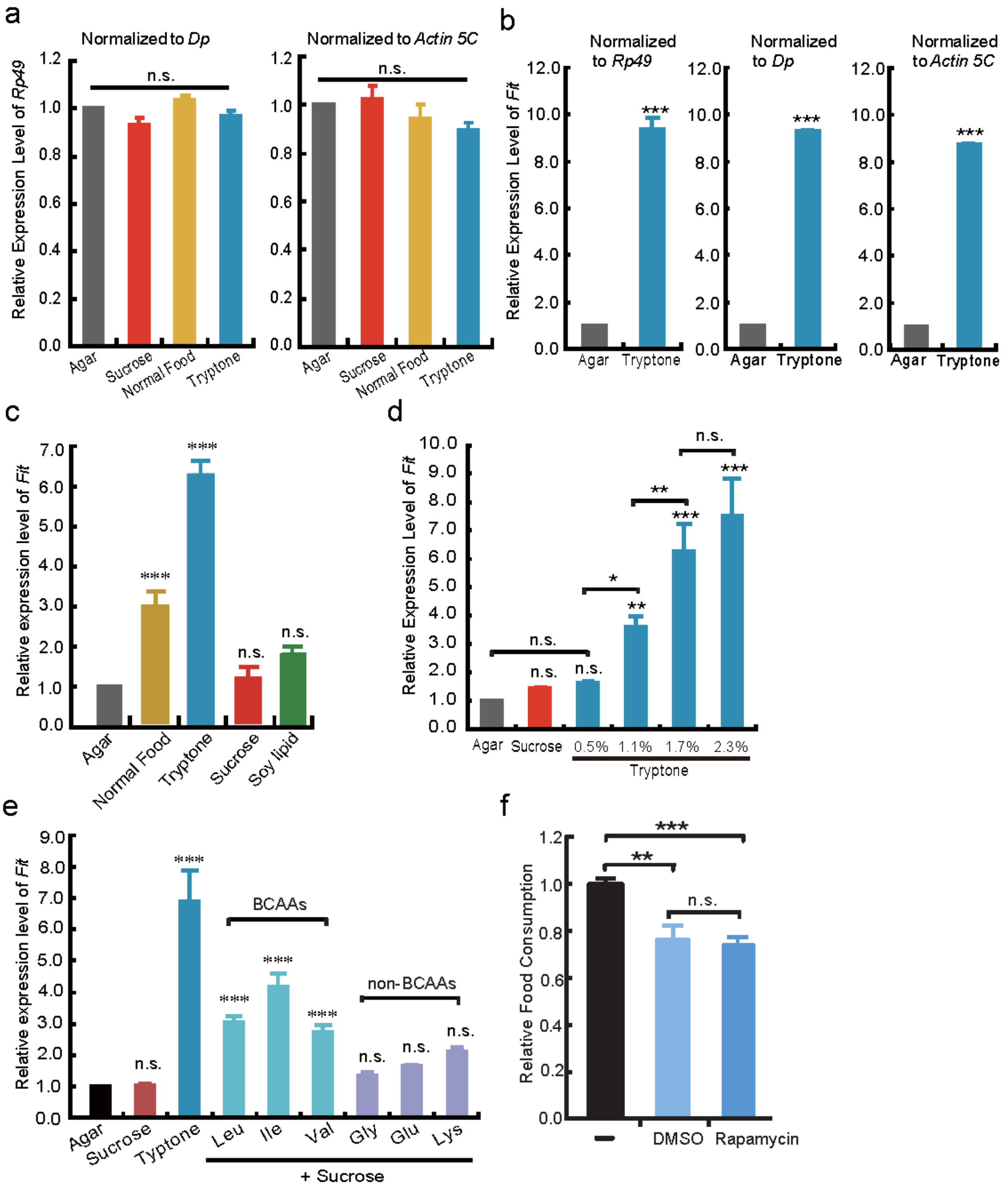


**Supplementary Figure 2 | Protein intake up-regulates *fit* expression level.**

(a) The expression levels of *Rp49* are comparable among starvation group (Agar) and three feeding groups. *Dp* and *Actin 5C* were used as the internal controls, respectively. n=3.

(b) *Fit* expression levels in WT flies greatly increased after 30 min feeding of tryptone, when analyzed with the *Rp49*, *Dp*, or *Actin 5C* as the internal control. n = 3.

(c) *Fit* expression levels significantly increased after the consumption of normal food or tryptone, but not after sucrose or lipid intake. n = 3.

(d) *Fit* expression levels increased after 30 min feeding of tryptone in a concentration-dependent manner. n = 3.

(e) BCAAs promote *Fit* expression, while non-BCAAs have little effect. Sucrose served as the sweetener, showing no effect on *Fit* expression on its own. n = 3.

(f) Food consumption was decreased when DMSO was added into the food, while it did not further decrease when Rapamycin was added. Sucrose-tryptone-mixed food was used. n = 4.

Data were analyzed by unpaired Student's *t* test in b and by one-way ANOVA, LSD's post hoc test in other panels. *, $p < 0.05$; **, $p < 0.01$; ***, $p < 0.001$. n.s. indicates no statistical significance.

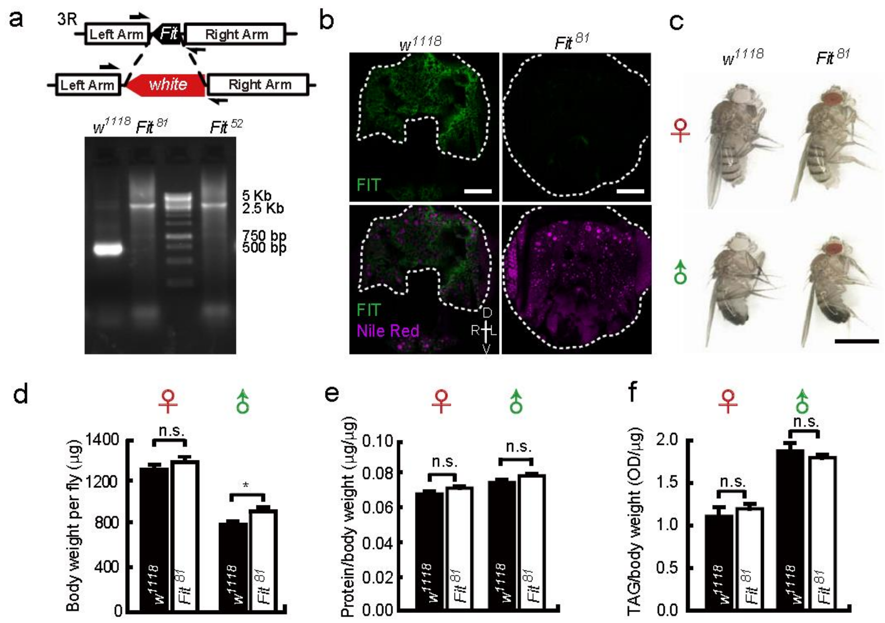


**Supplementary Figure 3 | *Fit* KO flies develop normally.**

(a) *Fit* KO flies were generated by homologue recombination. Two genomic fragments adjacent to the *Fit* gene were cloned as left (3.4 Kb) and right (5.4 Kb) arms into the vector pEndsOut-Riko flanking the *white* gene (upper panel). KO candidates were selected based on red-eye phenotype, and screened by PCR (lower panel) using the primers shown by arrows in the upper panel.

(b) FIT antibody detected FIT expression in head section of WT but not $Fit^{81}$ mutant flies. The FB region on the head section is circled by dashed lines. Scale bar, 100 µm.

(c) Representive images of $Fit^{81}$ mutant and WT control $w^{1118}$ flies. Scale bar, 1 mm.

(d, e and f) Body weight (d, n = 15), protein content (e, n = 9), and lipid content (f, n = 6) are comparable between $Fit^{81}$ mutant and WT flies in both sexes.

Data were analyzed by unpaired Student's *t* test in d, e, f. *, $p < 0.05$; n.s. indicates no statistical significance.

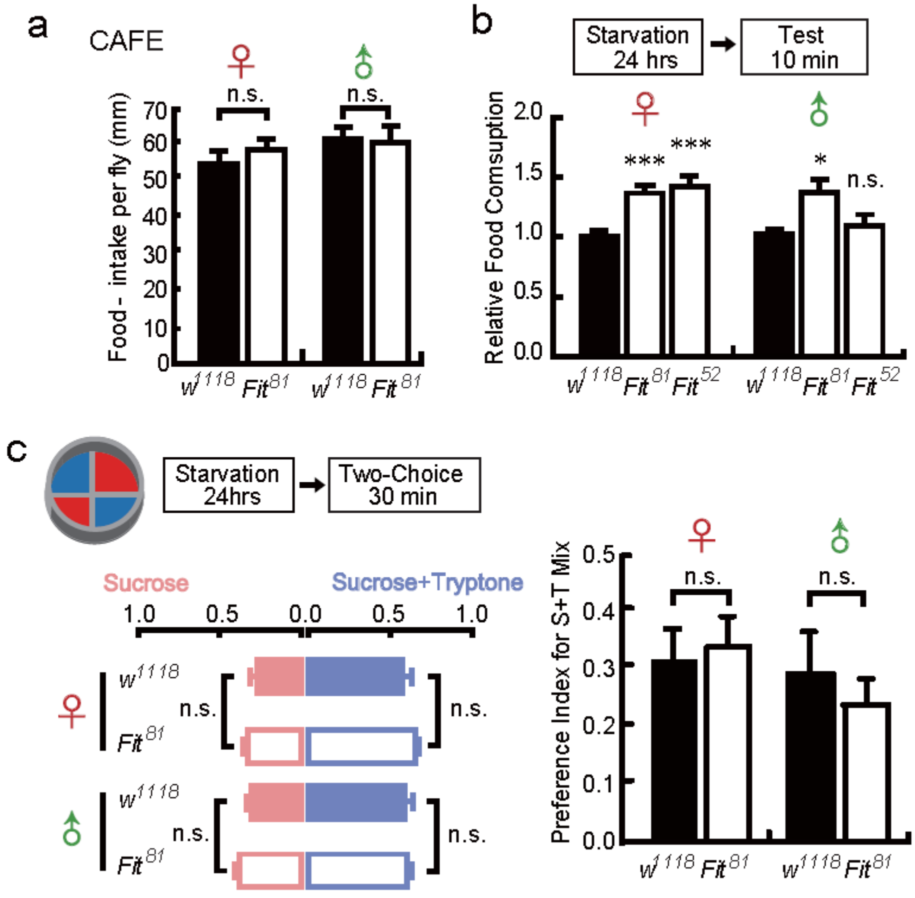


**Supplementary Figure 4 | Feeding behavior in *Fit* KO flies.**

(a,,b) Compared to WT control flies, *Fit* KO flies showed normal basal feeding (a, n = 20-28), but exhibited increased food intake after starvation (b, n = 9-19).

(c) In the modified two-choice feeding assay, *Fit* KO and $w^{1118}$ flies showed similar preference for the tryptone-sucrose mix versus sucrose only. Left panel shows the Choice Ratio (CR) for two nutrients. Right panel shows the Preference Index calculated as $CR_{S+T}$ – $CR_S$. n = 6.

Data were analyzed by unpaired Student's *t* test in a, c and by one-way ANOVA, LSD's post hoc test in b. *, p < 0.05; ***, p < 0.001. n.s. indicates no statistical significance.

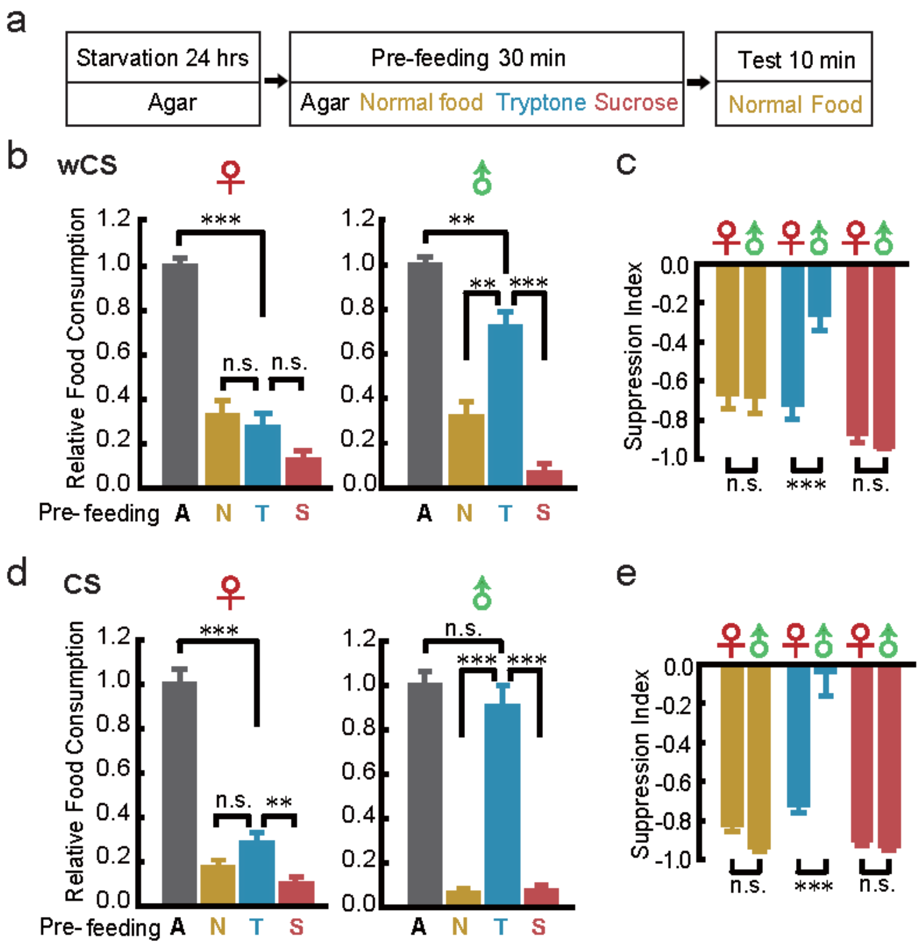


**Supplementary Figure 5 | Feeding behavior of two WT fly strains in the pre-feeding assay.**

In pre-feeding assay (a), WT flies of wCS (b, c) and CS (d, e) displayed significant differences between sexes in tryptone pre-feeding groups, but little sexual difference in normal food or sucrose pre-feeding groups. n = 8-10.

Data were analyzed by One-Way ANOVA in b, d and by two-way ANOVA, Bonferroni test in c, e. P(Food*Sex) = 4.01E-4 (two-way ANOVA, Bonferroni test) in c. P(Food*Sex) = 4.43E-10 (two-way ANOVA, Bonferroni test) in e. *, $p < 0.05$; **, $p < 0.01$; ***, $p < 0.001$. n.s. indicates no statistical significance.

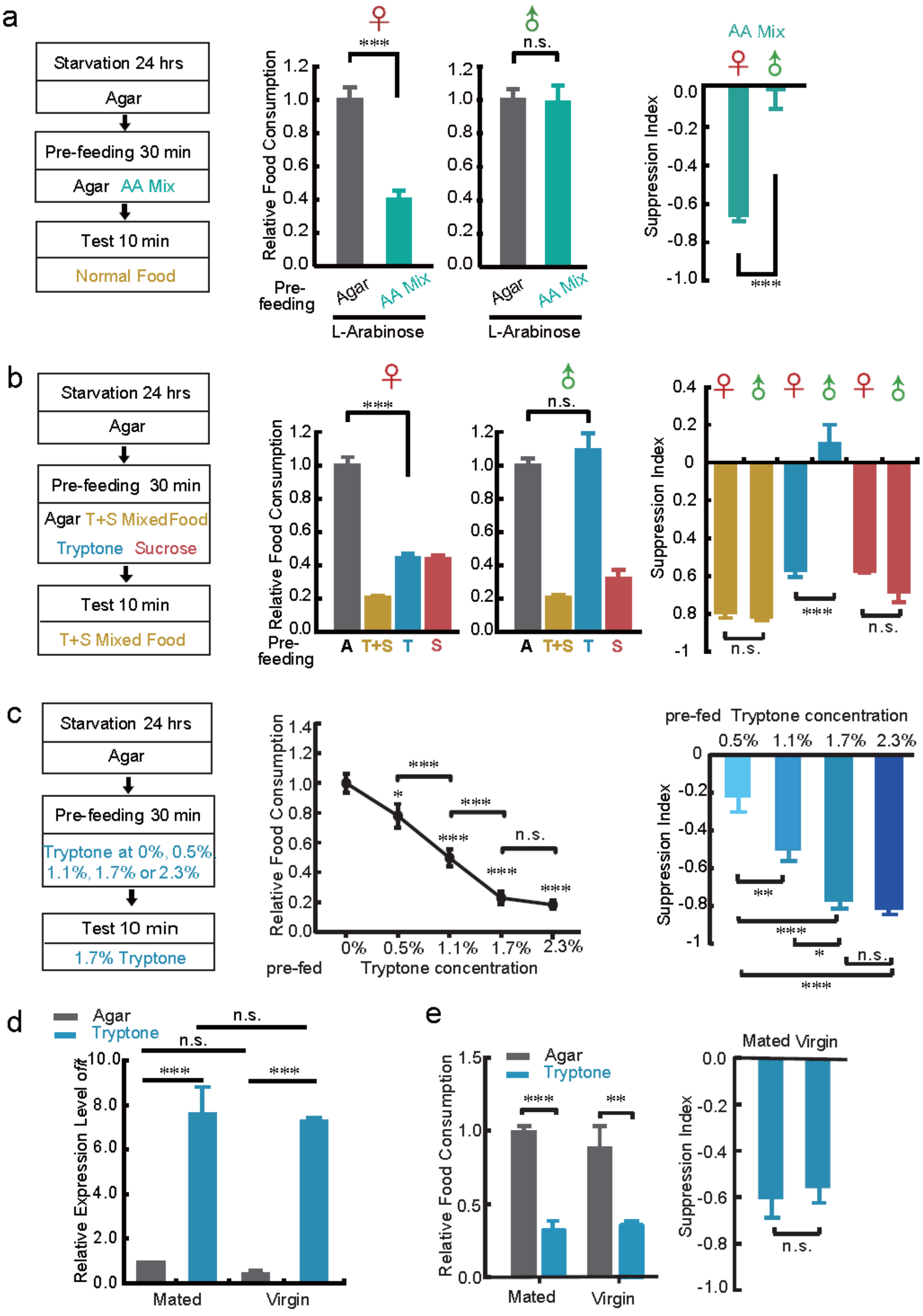


**Supplementary Figure 6 | WT $w^{1118}$ flies show sexual differences in modified pre-feeding assay.**

(a) Pre-feeding of AA mix significantly suppressed subsequent feeding in female flies. n = 6. Data were analyzed by unpaired Student's *t* test.

(b) When tryptone-sucrose mixed food (T+S mixed food) was used instead of normal food in test feeding phase, WT flies also exhibited significant sexual differences in the tryptone pre-feeding groups. n = 8. Data were analyzed by one-way ANOVA, LSD's post hoc test in Food Consumption experiments. For Suppression Index analysis, P (Food*Sex) =1.31E-9 (two-way ANOVA, Bonferroni test).

(c) After pre-feeding of tryptone at different concentration, flies exhibited gradually decreased tryptone intake in the test feeding. n = 6. One-way ANOVA, LSD's post hoc test.

(d) *Fit* expression levels were comparable between mated and virgin females. n = 4. P (Food*Mated) = 0.8615 (two-way ANOVA, Bonferroni test).

(e) Mated and virgin females exhibited similar PIFI effect, and the SI are comparable between them. n = 7-9. P (Food*Mated) = 0.3159 (two-way ANOVA, Bonferroni test). For Suppression Index analysis, data were analyzed by unpaired Student's *t* test.

*, $p < 0.05$; **, $p < 0.01$; ***, $p < 0.001$. n.s. indicates no statistical significance.

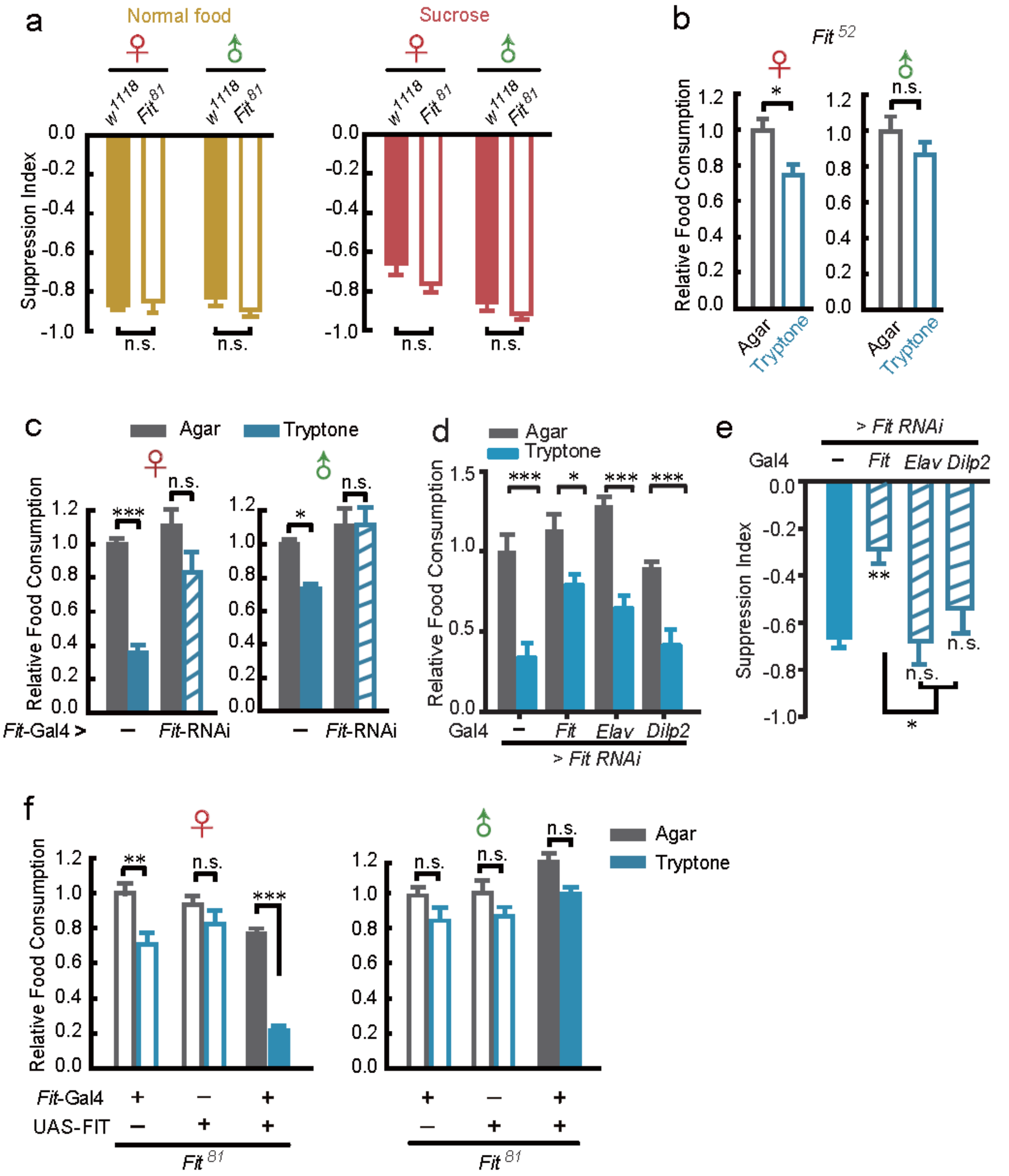


**Supplementary Figure 7 | *Fit* mutant flies are deficient in protein feeding.**

(a) The suppression indexes of normal food and sucrose were comparable between $Fit^{81}$ mutant and WT flies. n = 8-9. Data were analyzed by unpaired Student's *t* test.

(b, c) The suppressive effect of tryptone pre-feeding was significantly reduced in $Fit^{52}$

mutant flies (b) and flies with FB-knock down of *Fit* (c). n = 7-11. Data were analyzed by unpaired Student's *t* test in b and two-way ANOVA, Bonferroni test in c. P (Food*Genotype) = 0.017 in female and 0.0948 in male (c).

(d, e) Knock down of *Fit* in the FB (with *Fit*-Gal4), but not in the nervous system (with *Elav*-Gal4 or *Dilp2*-Gal4), resulted in a significant reduction in the PIFI effect. n = 8-9. P (Food*Genotype) =0.0108 (two-way ANOVA, Bonferroni test) in d. Data were analyzed by one-way ANOVA, LSD's post hoc test in e.

(f) The reduction in feeding suppression observed in $Fit^{81}$ female flies was rescued by FB-expression of FIT. n = 6-12. P (Food*Genotype)=0.0002 in female and 0.885 in male (two-way ANOVA, Bonferroni test).

*, p < 0.05; **, p < 0.01; ***, p < 0.001. n.s. indicates no statistical significance.

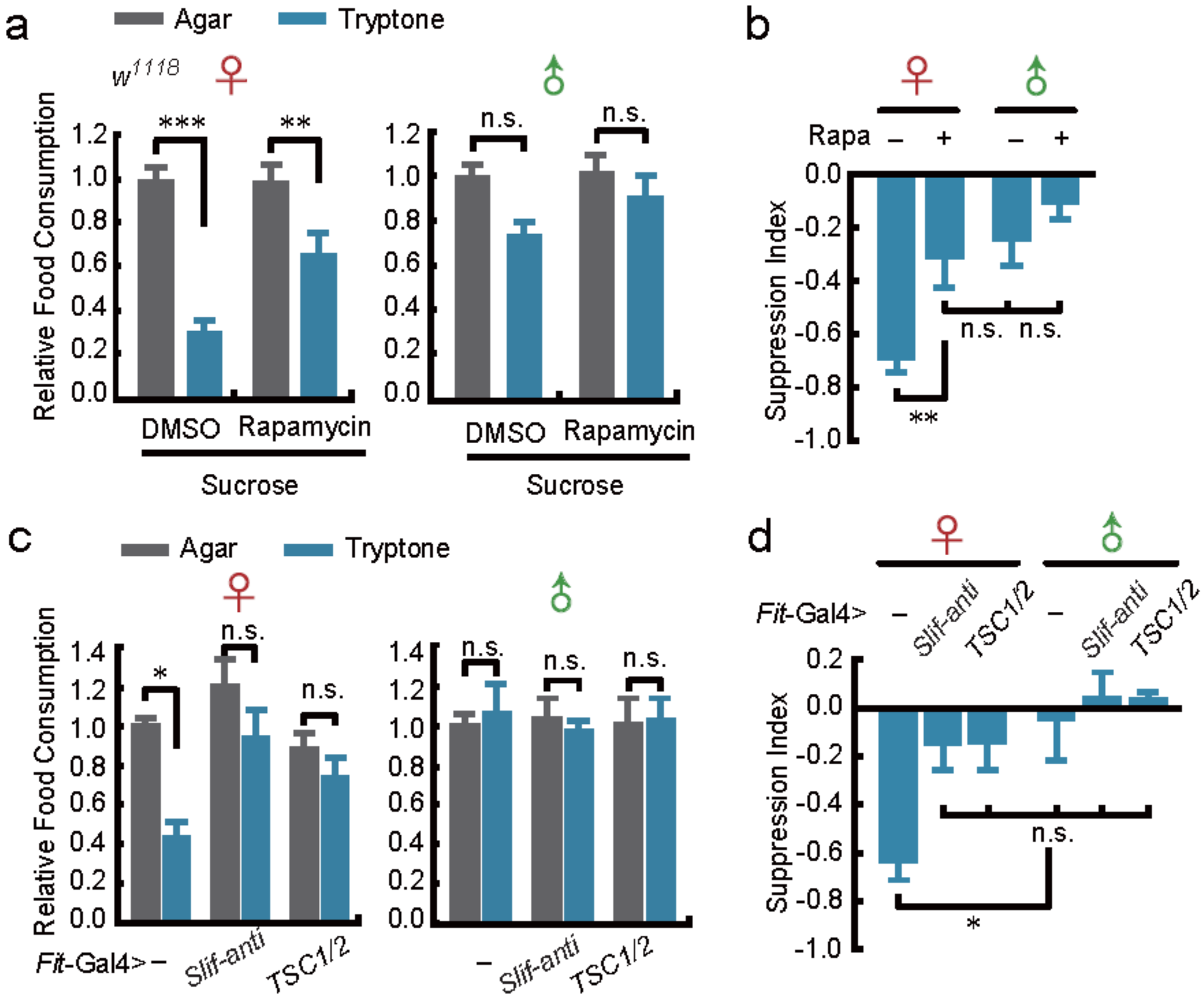


**Supplementary Figure 8 | The feeding suppression effect of tryptone in WT female flies is diminished following blocking of the AA sensing pathway**

(a, b) Upon Rapamycin treatment, the suppressive effect of tryptone was significantly reduced. n = 9-10. Sucrose served as the sweetener. P (Drug*Sex) = 0.0086 in female and 0.3511 in male (two-way ANOVA, Bonferroni test) in a. Data were analyzed by one-way ANOVA, LSD's post hoc test in b.

(c, d) The suppressive effect of tryptone pre-feeding was abolished in female flies when AA transporter *Slif* was knocked down (with *Slif*-anti), or when the negative regulator of

TOR pathway TSC1/2 was overexpressed in the FB. $n = 7\text{-}10$. P (Food*Genotype) = 0.1754 in female and 0.8155 in male (two-way ANOVA, Bonferroni test) in c. Data were analyzed by one-way ANOVA, LSD's post hoc test in d.

*, $p < 0.05$; **, $p < 0.01$; ***, $p < 0.001$. n.s. indicates no statistical significance.

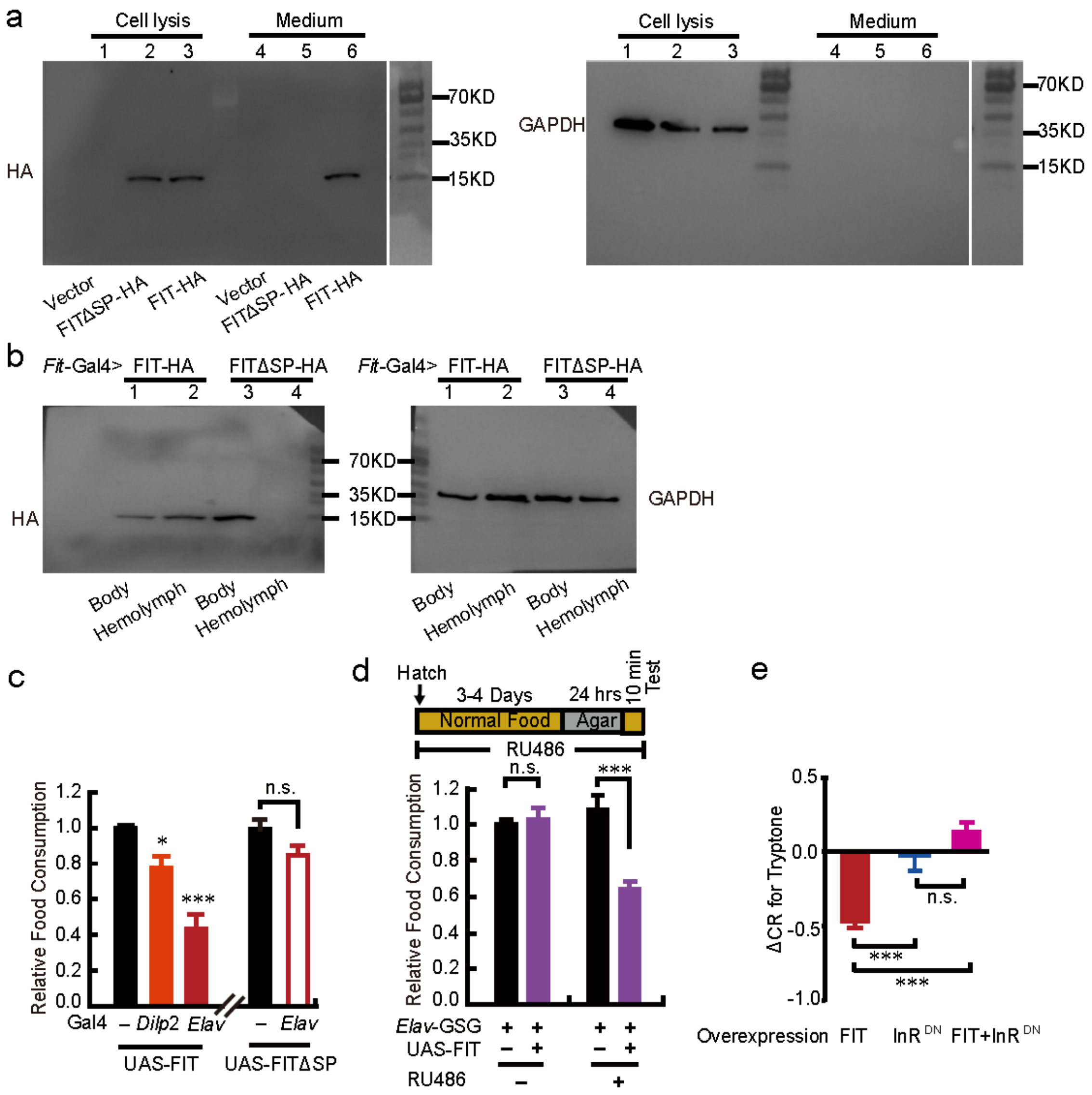


**Supplementary Figure 9 | Temporary expression of FIT in the nervous system suppresses feeding behavior.**

(a, b) The full size pictures of Western gels. HA signals were detected in medium (a) or in fly hemolymph (b) when FIT-HA was expressed, not however when FITΔSP-HA was expressed.

(c) Ectopic expressing FIT in the nervous system suppressed feeding, whereas expression of FITΔSP showed no such effect. n = 8-16. Data were analyzed by unpaired Student's *t*

test.

(d) RU486-induced neuronal expression of FIT suppressed food consumption. n = 8. P (Drug*Genotype) = 0.0003 (two-way ANOVA, Bonferroni test).

(e) The difference between overexpression groups and their parental controls showing as ΔCR for Tryptone = $(CR_{Overexpression} - CR_{control})/CR_{control}$. Overexpression of FIT resulted in a significant decease in the CR for tryptone, while overexpression of $InR^{DN}$ or FIT together with $InR^{DN}$ did not affect this choice ratio.

One-way ANOVA, LSD's post hoc test. *, $p < 0.05$; ***, $p < 0.001$. n.s. indicates no statistical significance.

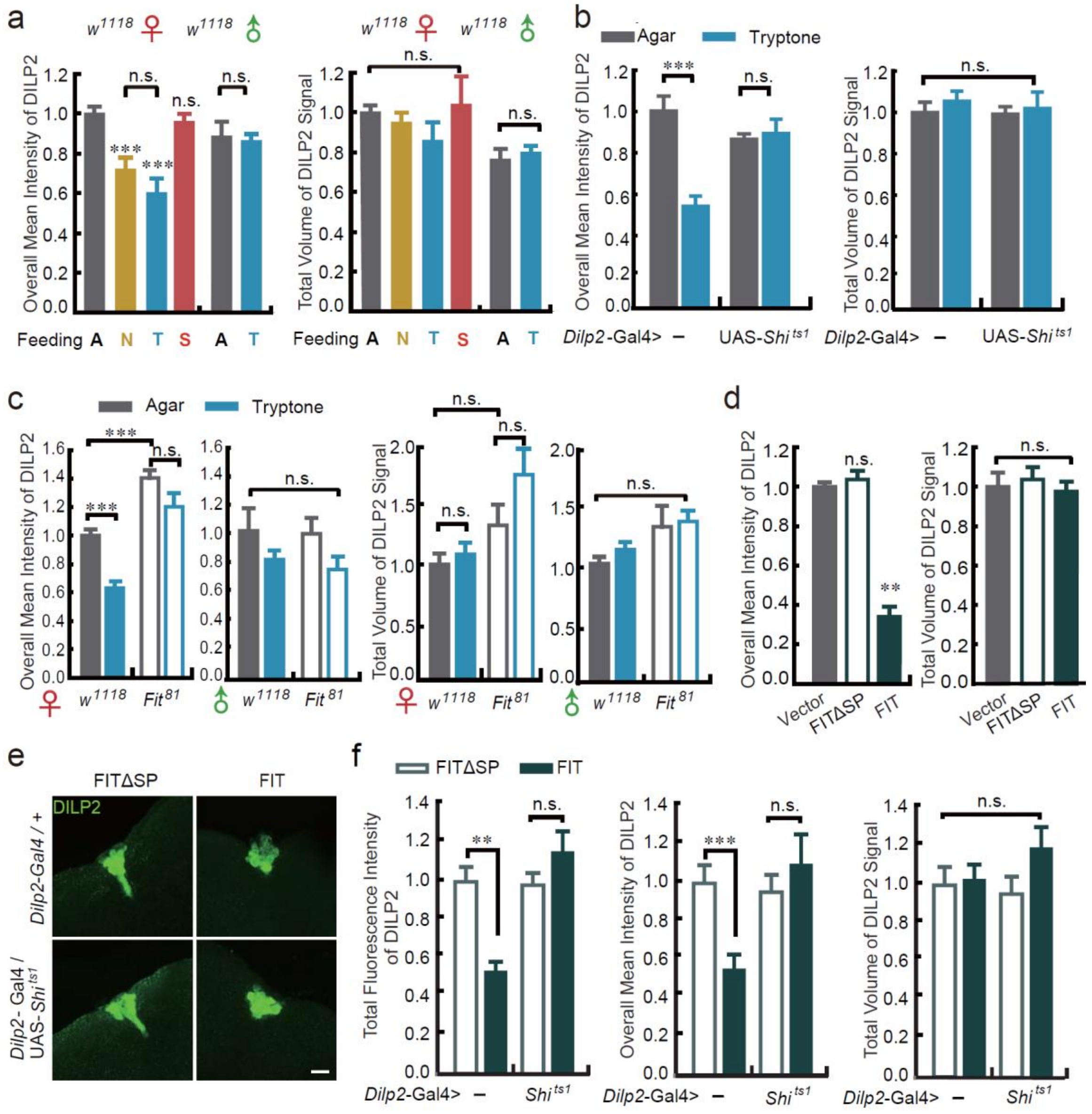

**Supplementary Figure 10 | Quantification of DILP2 immunostaining signals.**

(a) Compared to that in agar (A) group, the overall mean of DILP2 signals were reduced in female flies after feeding of normal food (N) or tryptone (T), but not after sucrose (S) feeding, while it was not reduced in male flies after tryptone feeding. The total volume remained unchanged in all groups. n = 7-49. P (Food*Sex) = 0.0494 in Overall Mean Intensity of DILP2 and 0.0737 in Total Volume of DILP2 Signal (two-way ANOVA,

Bonferroni test).

(b) The overall mean of DILP2 signals did not reduce after tryptone feeding in female flies with the secretion of IPCs blocked. The total volume remained unchanged. n = 12-16. P (Food*Genotype) = 3.49E-5 in Overall Mean Intensity of DILP2 and 0.8331 in Total Volume of DILP2 Signal (two-way ANOVA, Bonferroni test).

(c) The overall mean of DILP2 signals did not reduce after tryptone feeding in $Fit^{81}$ mutant female and male flies. The total volume remained unchanged. n = 19-33. P (Food*Genotype) = 0.1647 in female and 0.7978 in male in Overall Mean Intensity of DILP2 (two-way ANOVA, Bonferroni test). P (Food*Genotype) = 0.2075 in female and 0.5294 in male in Total Volume of DILP2 Signal (two-way ANOVA, Bonferroni test).

(d) The overall mean of DILP2 signals significantly reduced in brains incubated with FIT- but not FITΔSP- conditioned medium. The total volume remained unchanged. n = 22-47. One-way ANOVA, LSD's post hoc test.

(e, f) Expressing $Shi^{ts1}$ in IPCs abolishes the reduction of DILP2 signal induced by FIT medium incubation. n = 13-14. Scale bar, 20 μm. P (Medium*Genotype) = 0.0018 in Total Fluorescence Intensity, 1.80E-5 in Overall Mean Intensity of DILP2 and 0.2493 in Total Volume of DILP2 Signal (two-way ANOVA, Bonferroni test).

**, p < 0.01; ***, p < 0.001. n.s. indicates no statistical significance.

**Supplementary Table 1 | The composition of amino acid mixture**

| Amino Acids | Ala | Arg | Asp | Cys | Glu | Gly | IIe | Leu | Lys |
|---|---|---|---|---|---|---|---|---|---|
| g/L | 0.53 | 0.94 | 1.24 | 0.04 | 2.99 | 0.34 | 0.43 | 1.17 | 1.22 |

| Amino Acids | Met | Phe | Pro | Ser | Thr | Trp | Tyr | Val | SUM |
|---|---|---|---|---|---|---|---|---|---|
| g/L | 0.35 | 0.58 | 1.36 | 0.22 | 0.32 | 0.18 | 0.53 | 0.93 | 13.36 |

**Supplemental Table 2丨Primers for gene cloning or qPCR analysis**

| Description | Primers |
|---|---|
| Cloning the coding sequences of FIT for UAS-FIT-HA flies | *Fit-cDNA-s-EcoRI* GGAATTCCATGAACTCAACTCTGGTGATTCTACTTC |
| | *Fit-cDNA-as-XbaI* GCTCTAGAGCTTACATTCCATTCGCCTGGCTCATTTG |
| Cloning the coding sequences of FITΔSP for UAS-FITΔSP-HA flies | *Fit-cDNA-Δ SP-EcoRI-B-F* CGGAATTCATTCGTTGGTCTGAGGAGGA |
| | *Fit-cDNA-as-XbaI* GCTCTAGAGCTTACATTCCATTCGCCTGGCTCATTTG |
| Cloning the genome sequence of *Fit* promoter for *Fit*-GAL4 flies | *Fit-pro-s-StuI* AAGGCCTTCGGACCTGTTTCGTTCATAAAAAAGATC |
| | *Fit-pro-as—EcoRI* GGAATTCCTTCAACGATCGCTGTTAAACTGTGC |
| Cloning the genome sequences of *Fit* left arm for *Fit* knock-out flies | *Fit-KO-left-s-SacII* TCCCCGCGGGACCTTGATTACTGAGCACAGATGG |
| | *Fit-KO-left-as-KpnI* GGGGTACCCCGTAAGCTCACGACTTGGTGATCAG |
| Cloning the genome sequences of *Fit* right arm for *Fit* knock-out flies | *Fit-KO-right-s-SpeI* GACTAGTCAACGATCGCTGTTAAACTGTGCC |
| | *Fit-KO-right-as-AscI* AGGCGCGCCACGAGCAGGAGTGTGGTTTGAAC |
| Screen primers for *Fit* knock-out flies | *Fit-KO-exleft-F* ATCGATTCAAGGAGCAGCTC |
| | *Fit-KO-exright-R* |

| | |
|---|---|
| | CTGAGTAGCAACATTGTGAGG |
| Cloning FIT-HA into pcDNA3.1 vector for cell culture | *Fit-pcDNA3.1-EcoRI-B-F* CGGAATTCATGAACTCAACTCTGGTGATTCTA |
| | *Fit-pcDNA3.1-HA-XhoI-B-R* CCGCTCGAGCTATGCGTAATCTGGAACATCG |
| Cloning FITΔSP-HA into pcDNA3.1 vector for cell culture | *Fit- pcDNA3.1-ΔSP-EcoRI-B-F* CGGAATTCATTCGTTGGTCTGAGGAGGA |
| | *Fit-pcDNA3.1-HA-XhoI-B-R* CCGCTCGAGCTATGCGTAATCTGGAACATCG |
| qPCR primers for *Fit* | *Fit-qPCR-F* TTGGTGCAGGCCAGGAATAT |
| | *Fit-qPCR-R* CACAGGGCCAGTTGACAGAGT |
| qPCR primers for *Dilp2* | *dilp2-qPCR-F* GTATGGTGTGCGAGGAGTAT |
| | *dilp2-qPCR-R* TGAGTACACCCCCAAGATAG |
| qPCR primers for *Rp49* | *rp49-qPCR-F* AGGGTATCGACAACAGAGTG |
| | *rp49-qPCR-R* CACCAGGAACTTCTTGAATC |
| qPCR primers for *CG8147* | *CG8147-qPCR-F* CAAAGGCGTGACTGATTCCA |
| | *CG8147-qPCR-R* GATTGCGGCCATCCAAAC |
| qPCR primers for *CG14688* | *CG14688-qPCR-F* ACGGAAATGTGGTGCACAAG |
| | *CG14688-qPCR-R* GCGTGGCGACTCTTTTGC |
| qPCR primers for *CG14867* | *CG14867-qPCR-F* TCAGCGACAGCGTCCAAA |
| | *CG14867-qPCR-R* GGATTTCCCGTATTGTTCTGGTT |
| qPCR primers for *CG30431* | *CG30431-qPCR-F* CCCGAGTGCGAAAAGAAGTT |
| | *CG30431-qPCR-R* GGCTGTCATGTGCAGCTTCA |
| qPCR primers for *Sug* | *Sug-qPCR-F* TCGGTGCGCTGCGTTATA |
| | *Sug-qPCR-R* |

| | |
|---|---|
| | CGCCGTTCCAAGTCCAAT |
| qPCR primers for *CG5773* | *CG5773-qPCR-F*<br>TGTTTTGCTCGCCGGATT |
| | *CG5773-qPCR-R*<br>ACGGGTGCCGCATTCA |
| qPCR primers for *CG6129* | *CG6129-qPCR-F*<br>AGCTGCGGAGACTATGACAACA |
| | *CG6129-qPCR-R*<br>TCCGGGTCCACATCAATTG |
| qPCR primers for *CG7227* | *CG7227-qPCR-F*<br>GCCAGTCCCAGGTGCAAGT |
| | *CG7227-qPCR-R*<br>CAGCGGTATTGGATGGAATTC |
| qPCR primers for *CG9451* | *CG9451-qPCR-F*<br>TCAGATGCCCCGCTTCTC |
| | *CG9451-qPCR-R*<br>TTTGCGGGTTTTGATGCA |
| qPCR primers for *CG13607* | *CG13607-qPCR-F*<br>CGTGGCTAGTGGTAAGGGATCA |
| | *CG13607-qPCR-R*<br>ACGGTGGTGCAGGTCACAT |
| qPCR primers for *CG15282* | *CG15282-qPCR-F*<br>TTCTTGGTGATCGTTTTTGTGGC |
| | *CG15282-qPCR-R*<br>CACCGAATCC TCCGAATCCA |
| qPCR primers for *Lsp2* | *Lsp2-qPCR-F*<br>TCCGGCTATGCCAGTAACCT |
| | *Lsp2-qPCR-R*<br>GATTCTCCCGGTGCCATTG |
| qPCR primers for *CG12116* | *CG12116-qPCR-F*<br>CGGTGGCGCTCAATCAG |
| | *CG12116-qPCR-R*<br>TCCACTCGCTCCAAGTGCTT |

**Supplementary Table 3**

| Figure | Genotype | Sex | Assays | Antibodies | Statistic Methods |
|---|---|---|---|---|---|
| **Figure 1a** | ***w*$^{1118}$** | **Female** | **Pre-feeding Assay: Starvation (Agar)→ Pre-feeding (Tryptone)→ Test (Tryptone or Sucrose)** | **-** | **Two-Way ANOVA<br>10min vs 0min: p = 1.01E-14, 20min vs 0min: p = 1.40E-21, 30min vs 0min: p = 3.39E-24.** |
| **Figure 1b** | ***w*$^{1118}$** | **Female** | **qPCR** | **-** | **-** |
| **Figure 1c** | ***w*$^{1118}$** | **Female** | **qPCR** | **-** | **Two-Way ANOVA<br>30min vs 0min in Tryptone: p = 2.03E-5, 60min vs 0min in Tryptone: p = 1.56E-4.** |
| **Figure 1d** | ***w*$^{1118}$** | **Female** | **qPCR** | **-** | **One-Way ANOVA<br>Trptone vs Agar: p = 2.13E-4, Agar with L-Arabinose vs Agar: p = 0.62, AA Mix vs Agar: p = 0.002, BCAAs vs Agar: p = 0.012, Trptone vs AA Mixr: p = 0.48.** |
| **Figure 1e** | ***w*$^{1118}$** | **Female** | **qPCR** | **-** | **Two-Way ANOVA<br>Tryptone vs Agar in DMSO: p = 4.34E-4, Tryptone vs Agar in Rapamycin: p = 0.90.** |
| **Figure 2a** | ***Fit*-Gal4 */UAS-GFP*** | **Female and Male** | ***Fit* expression pattern** | **-** | **-** |
| **Figure 2b** | ***Fit*-Gal4 / *UAS-GFP*** | **Female** | **Immunohistochemical stain ing** | **Nile Red** | **-** |
| **Figure 2c** | ***Fit*-Gal4 / *UAS-GFP*** | **Female and Male** | **Immunohistochemical stain ing** | **anti-FIT, Nile Red** | **-** |
| **Figure 2d** | ***Fit*-Gal4 / *UAS-GFP*** | **Female and** | **Qualification of staining signal** | **anti-FIT, Nile Red** | **One-Way ANOVA<br>female fed with Protein Food vs male fed with** |

| | | | | | |
|---|---|---|---|---|---|
| | | **Male** | | | **Protein Food: p = 7.86E-5, female fed with Protein Food vs female fed without Protein Food: p = 2.06E-6.** |
| **Figure 2e** | ***w*[1118]** | **Female and Male** | **qPCR** | **-** | **Two-Way ANOVA**<br>**femle: head vs body: p = 1.06E-4, fat body vs body: p = 1.56E-4.**<br>**male: head vs body: p = 0.03, fat body vs body: p = 7.23E-3.** |
| **Figure 3a** | **-** | **-** | **Pre-feeding Assay: Starvation (Agar)→ Pre-feeding (Agar, Normal Food, Tryptone, Sucrose)→ Test (Tryptone or Sucrose)** | **-** | **-** |
| **Figure 3b** | ***w*[1118]** | **Female and Male** | **Pre-feeding Assay, as Figure 3a** | **-** | **One-Way ANOVA**<br>**female: Normal Food vs Agar: p = 1.17E-12, Tryptone vs Agar: p = 2.43E-10, Sucrose vs Agar: p = 9.30E-10, Normal Food vs Tryptone: p = 0.053, Tryptone vs Sucrose: p = 0.64.**<br>**male: Normal Food vs Agar: p = 4.15E-11, Tryptone vs Agar: p = 0.094, Sucrose vs Agar: p = 1.96E-11, Normal Food vs Tryptone: p = 2.37E-8, Tryptone vs Sucrose: p = 1.08E-7.** |
| **Figure 3c** | ***w*[1118]** | **Female and Male** | **Pre-feeding Assay, as Figure 3a** | **-** | **Two-Way ANOVA** |
| **Figure 3d** | ***Fit*[81]** | **Female and Male** | **Pre-feeding Assay, as Figure 3a** | **-** | **One-Way ANOVA**<br>**female: Normal Food vs Agar: p = 1.56E-12, Tryptone vs Agar: p = 0.0013, Sucrose vs Agar: p =3.13E-11.**<br>**male: Normal Food vs Agar: p =8.02E-15, Tryptone vs Agar: p = 0.059, Sucrose vs Agar: p = 3.32E-15.** |

| | | | | | |
|---|---|---|---|---|---|
| Figure 3e | $w^{1118}$<br>$Fit^{81}$ and $Fit^{52}$ | Female and Male | Pre-feeding Assay, as Figure 3a | - | One-Way ANOVA<br>$w^{1118}$ female vs $Fit^{81}$ female: p = 0.0068, $w^{1118}$ female vs $Fit^{81}$ female: p = 0.0019, $w^{1118}$ female vs $w^{1118}$ male: p = 0.0017, $w^{1118}$ female vs $Fit^{81}$ male: p = 0.0015, $w^{1118}$ female vs $Fit^{52}$ male: p = 0.0017. |
| Figure 3f | *Fit*-Gal4/+<br>*Fit*-Gal4/+;*Fit*-RNAi/+ | Female and Male | Pre-feeding Assay, as Figure 3a | - | Unpaired Student *t* test<br>female: *Fit*-Gal4/+ vs *Fit*-Gal4>UAS-Fit-RNAi: p = 9.07E-5.<br>male: *Fit*-Gal4/+ vs *Fit*-Gal4>UAS-Fit-RNAi : p = 0.19. |
| Figure 3g | *Fit*-Gal4/+; $Fit^{81}$/ $Fit^{81}$<br>UAS-FIT/+; $Fit^{81}$/ $Fit^{81}$<br>*Fit*-Gal4/UAS-FIT ;$Fit^{81}$/ $Fit^{81}$ | Female and Male | Pre-feeding Assay, as Figure 3a | - | One-Way ANOVA<br>female:<br>*Fit*-Gal4;$Fit^{81}$/$Fit^{81}$ vs *Fit*-Gal4/UAS-FIT;$Fit^{8}$/$Fit^{811}$ : p = 4.93E-6,<br>UAS-FIT;$Fit^{81}$/$Fit^{81}$ vs *Fit*-Gal4/UAS-FIT;$Fit^{8}$/$Fit^{811}$ : p = 3.37E-7,<br>*Fit*-Gal4;$Fit^{81}$/$Fit^{81}$ vs UAS-FIT;$Fit^{8}$/$Fit^{811}$ : p = 0.12.<br>male:<br>*Fit*-Gal4;$Fit^{8}$/$Fit^{811}$ vs *Fit*-Gal4/UAS-FIT;$Fit^{8}$/$Fit^{811}$ :p = 0.78, UAS-FIT;$Fit^{81}$/$Fit^{81}$ vs *Fit*-Gal4/UAS-FIT;$Fit^{8}$/$Fit^{811}$ :p = 0.92, *Fit*-Gal4;$Fit^{81}$/$Fit^{81}$ vs UAS-FIT;$Fit^{8}$/$Fit^{811}$ : p = 0.86. |
| Figure 4a | $w^{1118}$<br>$Fit^{81}$ | Female | Two-choice feeding assay | - | Unpaired Student *t* test<br>Sucrose pre-feeding condition: Sucrose selection in $w^{1118}$ vs $Fit^{81}$: p = 0.57, Tryptone selection in $w^{1118}$ vs $Fit^{81}$: p = 0.51.<br>No pre-feeding condition: Sucrose selection in $w^{1118}$ vs $Fit^{81}$: p = 0.012, Tryptone selection in $w^{1118}$ vs $Fit^{81}$: p = 0.47. Tryptone pre-feeding condition: Sucrose selection in $w^{1118}$ vs $Fit^{81}$: p = 7.09E-4, Tryptone selection in $w^{1118}$ vs $Fit^{81}$: p = 3.70E-4. |

| | | | | | |
|---|---|---|---|---|---|
| **Figure 4b** | ***w*$^{1118}$**<br>***Fit*$^{81}$** | **Male** | **Two-choice feeding assay** | **-** | **Unpaired Student *t* test**<br>**Sucrose pre-feeding condition: Sucrose selection in *w*$^{1118}$ vs *Fit*$^{81}$: p = 0.90, Tryptone selection in *w*$^{1118}$ vs *Fit*$^{81}$: p = 0.61. No pre-feeding condition: Sucrose selection in *w*$^{1118}$ vs *Fit*$^{81}$: p = 0.72, Tryptone selection in *w*$^{1118}$ vs *Fit*$^{81}$: p = 0.11.**<br>**Tryptone pre-feeding condition: Sucrose selection in *w*$^{1118}$ vs *Fit*$^{81}$: p = 0.57, Tryptone selection in *w*$^{1118}$ vs *Fit*$^{81}$: p = 0.94.** |
| **Figure 4c** | ***Fit*-Gal4/+**<br>**+/UAS-FIT**<br>***Fit*-Gal4/UAS-FIT** | **Female** | **Two-choice feeding assay** | **-** | **One-Way ANOVA**<br>**Sucrose pre-feeding condition: Sucrose selection in *Fit*-Gal4/+ vs +/UAS-FIT: p = 0.82, Sucrose selection in *Fit*-Gal4/+ vs *Fit*-Gal4/UAS-FIT: p = 0.39, Sucrose selection in +/UAS-FIT vs *Fit*-Gal4/UAS-FIT: p = 0.28; Tryptone selection in *Fit*-Gal4/+ vs +/UAS-FIT: p = 0.31, Tryptone selection in *Fit*-Gal4/+ vs *Fit*-Gal4/UAS-FIT: p = 3.11E-9, Tryptone selection in +/UAS-FIT vs *Fit*-Gal4/UAS-FIT: p = 1.05E-8.**<br>**No pre-feeding condition: Sucrose selection in *Fit*-Gal4/+ vs +/UAS-FIT: p = 0.76, Sucrose selection in *Fit*-Gal4/+ vs *Fit*-Gal4>UAS-FIT: p = 0.0015, Sucrose selection in +/UAS-FIT vs *Fit*-Gal4/UAS-FIT: p = 0.0010; Tryptone selection in *Fit*-Gal4/+ vs +/UAS-FIT: p = 0.58, Tryptone selection in *Fit*-Gal4/+ vs *Fit*-Gal4/UAS-FIT: p = 0.0040, Tryptone selection in +/UAS-FIT vs *Fit*-Gal4/UAS-FIT: p = 0.0013.**<br>**Tryptone pre-feeding condition: Sucrose selection in *Fit*-Gal4/+ vs +/UAS-FIT: p = 0.56, Sucrose selection in *Fit*-Gal4/+ vs *Fit*-Gal4/UAS-FIT: p = 0.73, Sucrose** |

| | | | | | |
|---|---|---|---|---|---|
| | | | | | **selection in +/UAS-FIT vs *Fit*-Gal4/UAS-FIT: p = 0.81; Tryptone selection in *Fit*-Gal4/+ vs +/UAS-FIT: p = 0.36, Tryptone selection in *Fit*-Gal4/+ vs *Fit*-Gal4/UAS-FIT: p = 0.72, Tryptone selection in + /UAS-FIT vs *Fit*-Gal4/UAS-FIT: p = 0.58.** |
| **Figure 4d** | ***Fit*-Gal4/+<br>+/UAS-FIT<br>*Fit*-Gal4/UAS-FIT** | **Male** | **Two-choice feeding assay** | **-** | **One-Way ANOVA<br>Sucrose pre-feeding condition: Sucrose selection in *Fit*-Gal4/+ vs +/UAS-FIT: p=0.67, Sucrose selection in *Fit*-Gal4/+ vs *Fit*-Gal4/UAS-FIT: p = 0.24, Sucrose selection in +/UAS-FIT vs *Fit*-Gal4/UAS-FIT: p = 0.43; Tryptone selection in *Fit*-Gal4/+ vs +/UAS-FIT: p = 0.28, Tryptone selection in *Fit*-Gal4/+ vs *Fit*-Gal4/UAS-FIT: p = 2.27E-5, Tryptone selection in +/UAS-FIT vs *Fit*-Gal4/UAS-FIT: p = 1.84E-4.<br>No pre-feeding condition: Sucrose selection in *Fit*-Gal4/+ vs +/UAS-FIT: p = 0.78, Sucrose selection in *Fit*-Gal4/+ vs *Fit*-Gal4/UAS-FIT: p = 0.23, Sucrose selection in +/UAS-FIT vs *Fit*-Gal4/UAS-FIT: p = 0.35; Tryptone selection in *Fit*-Gal4/+ vs +/UAS-FIT: p = 0.84, Tryptone selection in *Fit*-Gal4/+ vs *Fit*-Gal4/UAS-FIT: p = 0.13, Tryptone selection in + /UAS-FIT vs *Fit*-Gal4/UAS-FIT: p = 0.19.<br>Tryptone pre-feeding condition: Sucrose selection in *Fit*-Gal4/+ vs +/UAS-FIT: p=0.78, Sucrose selection in *Fit*-Gal4/+ vs *Fit*-Gal4/UAS-FIT: p = 0.56, Sucrose selection in + /UAS-FIT vs *Fit*-Gal4/UAS-FIT: p = 0.76; Tryptone selection in *Fit*-Gal4/+ vs +/UAS-FIT: p = 0.39, Tryptone selection in *Fit*-Gal4/+ vs *Fit*-Gal4/UAS-FIT: p = 0.28, Tryptone selection in + /UAS-FIT vs *Fit*-Gal4/UAS-FIT: p = 0.82.** |
| **Figure** | **-** | **-** | **Signal peptide prediction in** | **-** | **-** |

| | | | | | |
|---|---|---|---|---|---|
| 5a | | | SignalP 4.1 | | |
| Figure 5b | - | - | Western Blot | HA, GAPDH | - |
| Figure 5c | *Fit*-Gal4/UAS-FIT-HA<br>*Fit*-Gal4/+;+/UAS-FITΔSP-HA | - | Western Blot | HA, GAPDH | - |
| Figure 5d | UAS-FIT /+<br>*Fit*-Gal4/UAS-FIT<br>UAS-FIT /+; +/*Lsp2*-Gal4<br>UAS-FIT /*Ppl*-Gal4<br>UAS-FITΔSP /+<br>*Ppl*-Gal4/+;+/UAS-FITΔSP-HA | - | Feeding Assay:<br>Starvation (Agar)→<br>Test (Normal Food) | - | Unpaired Student *t* test<br>+/UAS-FIT vs *Fit*-Gal4>UAS-FIT: p = 0.0025, +/UAS-FIT vs *Lsp2*-Gal4>UAS-FIT: p = 2.26E-4, +/UAS-FIT vs *Ppl*-Gal4>UAS-FIT: p = 2.01E-8, +/UAS-FITΔSP vs *Fit*-Gal4>UAS-FITΔSP: p = 0.25. |
| Figure 5e | *Elav*-Gal4/+<br>UAS-FIT /+<br>*Elav*-Gal4/+;+ /UAS-FIT<br>UAS-$InR^{DN}$ /+<br>*Elav*-Gal4/+;+ / UAS-$InR^{DN}$<br>UAS-FIT/ UAS-$InR^{DN}$<br>*Elav*-Gal4/+; UAS-FIT/ UAS-$InR^{DN}$ | - | Feeding Assay:<br>Starvation (Agar)→<br>Test (Normal Food) | - | Unpaired Student *t* test<br>+/UAS-FIT vs *Elav*-Gal4>UAS-FIT: p = 4.70E-5, +/UAS-$InR^{DN}$ vs *Elav*-Gal4>UAS-$InR^{DN}$: p = 0.015, UAS-FIT ;UAS- $InR^{DN}$ vs *Elav*-Gal4/+; UAS-FIT/UAS-$InR^{DN}$: p = 0.0028, *Elav*-Gal4>UAS-$InR^{DN}$ vs *Elav*-Gal4/+; UAS-FIT/UAS-$InR^{DN}$: p = 0.37. |
| Figure 5f | *Elav*-Gal4/+<br>UAS-FIT /+<br>*Elav*-Gal4/+;+ /UAS-FIT<br>UAS-$InR^{DN}$ /+<br>*Elav*-Gal4/+;+ / UAS-$InR^{DN}$<br>*UAS-Fit/* UAS-$InR^{DN}$<br>*Elav*-Gal4/+; UAS-FIT/ UAS-$InR^{DN}$ | - | Two Choice Assay:<br>Pre-feed (Sucrose)→<br>Test (Tryptone or Sucrose) | - | Unpaired Student *t* test<br>+/UAS-FIT vs *Elav*-Gal4>UAS-FIT: p = 1.23E-6, +/UAS-$InR^{DN}$ vs *Elav*-Gal4>UAS-$InR^{DN}$: p = 0.65, UAS-FIT; UAS-$InR^{DN}$ vs *Elav*-Gal4/+;UAS-FIT/ UAS-$InR^{DN}$: p = 0.26, *Elav*-Gal4>UAS-$InR^{DN}$ vs *Elav*-Gal4/+;UAS-FIT/UAS- $InR^{DN}$: p = 0.45. |
| Figure 6a | $w^{1118}$ | Female and Male | Immunohistochemical stain ing | DILP2 | - |
| Figure 6b | $w^{1118}$ | Female and Male | Signal intensity statistics of staining | DILP2 | Two-Way ANOVA<br>female: pN-A = 9.77E-6, pT-A = 1.57E-6, pS-A = 0.09.<br>male: pT-A = 0.06. |

| | | | | | |
|---|---|---|---|---|---|
| **Figure 6c** | **-** | **-** | **-** | **-** | **-** |
| **Figure 6d** | ***Dilp2*-Gal4/+**<br>***Dilp2*-Gal4/UAS-shi$^{ts1}$** | **Female** | **Immunohistochemical staining** | **DILP2** | **-** |
| **Figure 6e** | ***Dilp2*-Gal4/+**<br>***Dilp2*-Gal4/UAS-shi$^{ts1}$** | **Female** | **Signal intensity statistics of staining** | **DILP2** | **Two-Way ANOVA**<br>**Tryptone vs Agar in *Dilp2*-Gal4: p = 3.92E-5, Tryptone vs Agar in *Dilp2*-Gal4>UAS-*shits1*: p = 0.60.** |
| **Figure 7a** | $w^{1118}$<br>$Fit^{81}$ | **Female** | **Immunohistochemical staining** | **DILP2** | **-** |
| **Figure 7b** | $w^{1118}$<br>$Fit^{81}$ | **Female** | **Signal intensity statistics of staining** | **DILP2** | **One-Way ANOVA, Two-Way ANOVA**<br>**female: Tryptone vs Agar in $w^{1118}$: p = 0.045, Tryptone vs Agar in $Fit^{81}$: p = 0.15, Agar in $Fit^{81}$ vs Agar in $w^{1118}$: p = 2.78E-5. males: Tryptone vs Agar in $w^{1118}$ p = 0.87, Tryptone vs Agar in $Fit^{81}$ p = 0.52.** |
| **Figure 7c** | $w^{1118}$<br>$Fit^{81}$ | **Female** | **qPCR** | **DILP2** | **Unpaired Student *t* test** |
| **Figure 7d** | $w^{1118}$ | **Female** | **Immunohistochemical staining** | **DILP2** | **-** |
| **Figure 7e** | $w^{1118}$ | **Female** | **Signal intensity statistics of staining** | **DILP2** | **One-Way ANOVA** |
| **Figure 7f** | **-** | **-** | **-** | **-** | **-** |
| **Sup. Figure 1b** | $w^{1118}$ | **Female and Male** | **Pre-feeding Assay:**<br>**Starvation (Agar) → Pre-feeding (Normal Food, Tryptone, Sucrose) → Test (Normal Food, Tryptone, Sucrose).** | **-** | **-** |
| **Sup. Figure** | $w^{1118}$ | **Female** | **qPCR** | **-** | **One-Way ANOVA** |

| | | | | | |
|---|---|---|---|---|---|
| 2a,c, d, e | | | | | |
| Sup. Figure 2b | $w^{1118}$ | Female | qPCR | - | Unpaired Student *t* test |
| Sup. Figure 2f | $w^{1118}$ | Female | Pre-feeding Assay: Starvation (Agar) → Pre-feeding (Agar, Agar+DMSO, Agar+DMSO+Rapamycin) → Test (Normal Food) | | One-Way ANOVA |
| Sup. Figure 3a | $w^{1118}$<br>$Fit^{81}$<br>$Fit^{52}$ | Female and Male | PCR | - | - |
| Sup. Figure 3b | $w^{1118}$<br>$Fit^{81}$ | Female | Immunohistochemical staining | anti-FIT<br>Nile-Red | - |
| Sup. Figure 3d-f | $w^{1118}$<br>$Fit^{81}$ | Female | Body Weight per Fly (d)<br>Protein/ Body Weight (e)<br>TAG/Body Weight (f) | - | Unpaired Student *t* test |
| Sup. Figure 4a | $w^{1118}$<br>$Fit^{81}$ | Female and Male | CAFE | - | Unpaired Student *t* test |
| Sup. Figure 4b | $w^{1118}$<br>$Fit^{81}$<br>$Fit^{52}$ | Female and Male | Pre-feeding Assay:<br>Starvation (Agar)→<br>Test (Normal Food) | - | One-Way ANOVA |
| Sup. Figure 4c | $w^{1118}$<br>$Fit^{81}$ | Female and Male | Two-choice feeding assay | - | Unpaired Student *t* test |
| Sup. Figure | wCS | Female and | Pre-feeding Assay: Starvation (Agar) → | - | One-Way ANOVA |

| | | | | | |
|---|---|---|---|---|---|
| **5b** | | **Male** | **Pre-feeding (Agar, Normal Food, Tryptone, Sucrose) → Test (Normal Food)** | | |
| **Sup. Figure 5c** | **wCS** | **Female and Male** | **Pre-feeding Assay: Starvation (Agar) → Pre-feeding (Agar, Normal Food, Tryptone, Sucrose) → Test (Normal Food)** | **-** | **Two-Way ANOVA** |
| **Sup. Figure 5d** | **CS** | **Female and Male** | **Pre-feeding Assay: Starvation (Agar) → Pre-feeding (Agar, Normal Food, Tryptone, Sucrose) → Test (Normal Food)** | **-** | **One-Way ANOVA** |
| **Sup. Figure 5e** | **CS** | **Female and Male** | **Pre-feeding Assay: Starvation (Agar) → Pre-feeding (Agar, Normal Food, Tryptone, Sucrose) → Test (Normal Food)** | **-** | **Two-Way ANOVA** |
| **Sup. Figure 6a** | ***$w^{1118}$*** | **Female and Male** | **Pre-feeding Assay: Starvation (Agar) → Pre-feeding (Agar, AA Mix)→ Test (Normal Food)** | **-** | **Unpaired Student *t* test** |
| **Sup. Figure 6b** | ***$w^{1118}$*** | **Female and Male** | **Pre-feeding Assay: Starvation (Agar) → Pre-feeding (Agar, T+S Mixed Food, Tryptone, Sucrose) → Test (T+S Mixed Food)** | **-** | **Unpaired Student *t* test<br>Two-Way ANOVA** |
| **Sup. Figure 6c** | ***$w^{1118}$*** | **Female** | **Pre-feeding Assay: Starvation (Agar) → Pre-feeding (Tryptone at** | **-** | **One-Way ANOVA** |

| | | | | | |
|---|---|---|---|---|---|
| | | | 0%, 0.5%, 1.1%, 1.7%, or 2.3% ) → Test (Tryptone at 1.7%) | | |
| Sup. Figure 6d | *w*$^{1118}$ | Female: Mated and Virgin | qPCR | - | Two-Way ANOVA |
| Sup. Figure 6e-f | *w*$^{1118}$ | Female: Mated and Virgin | Pre-feeding Assay: Starvation (Agar)→ Pre-feeding (Agar, Tryptone)→ Test (Normal Food) | - | Unpaired Student *t* test |
| Sup. Figure 7a | *w*$^{1118}$<br>*Fit* $^{81}$ | Female and Male | Pre-feeding Assay: Starvation (Agar)→ Pre-feeding (Agar, Normal Food, Sucrose)→ Test (Normal Food) | - | Unpaired Student *t* test |
| Sup. Figure 7b | *Fit*$^{52}$ | Female and Male | Pre-feeding Assay : Starvation (Agar)→ Pre-feeding (Agar, Tryptone)→ Test (Normal Food) | - | Unpaired Student *t* test |
| Sup. Figure 7c | *Fit*-Gal4/+<br>*Fit*-Gal4/+;*Fit*-RNAi/+ | Female and Male | Pre-feeding Assay: Starvation (Agar)→ Pre-feeding (Agar, Tryptone)→ Test (Normal Food) | - | Two-Way ANOVA |
| Sup. Figure 7d | *Fit*-RNAi/+<br>*Fit*-Gal4/+;*Fit*-RNAi/+<br>*Elav*-Gal4/+;*Fit*-RNAi/+<br>*Dilp2*-Gal4/+;*Fit*-RNAi/+ | Female | Pre-feeding Assay: Starvation (Agar)→ Pre-feeding (Agar, Tryptone)→ | - | d: Two-Way ANOVA<br>e: One-Way ANOVA |

| | | | | | |
|---|---|---|---|---|---|
| | | | **Test (Normal Food)** | | |
| **Sup. Figure 7e** | ***Fit*-RNAi/+**<br>***Fit*-Gal4/+;*Fit*-RNAi/+**<br>***Elav*-Gal4/+;*Fit*-RNAi/+**<br>***Dilp2*-Gal4/+;*Fit*-RNAi/+** | **Female** | **Pre-feeding Assay: Starvation (Agar)→ Pre-feeding (Agar, Tryptone)→ Test (Normal Food)** | **-** | **One-Way ANOVA** |
| **Sup. Figure 7f** | ***Fit*-Gal4/+;*Fit*$^{81}$**<br>**UAS-FIT /+;*Fit*$^{81}$**<br>***Fit*-Gal4/ UAS-FIT ;*Fit*$^{81}$** | **Female and Male** | **Pre-feeding Assay: Starvation (Agar)→ Pre-feeding (Agar, Tryptone)→ Test (Normal Food)** | **-** | **Two-Way ANOVA** |
| **Sup. Figure 8a** | ***w*$^{1118}$** | **Female and Male** | **Pre-feeding Assay: Starvation (Agar)→ Pre-feeding (Agar, Tryptone)→ Test (Normal Food)** | **-** | **Two-Way ANOVA** |
| **Sup. Figure 8b** | ***w*$^{1118}$** | **Female and Male** | **Pre-feeding Assay: Starvation (Agar)→ Pre-feeding (Agar, Tryptone)→ Test (Normal Food)** | **-** | **One-Way ANOVA** |
| **Sup. Figure 8c** | ***Fit*-Gal4/ *slif-anti***<br>***Fit*-Gal4/*TSC1/2*** | **Female and Male** | **Pre-feeding Assay: Starvation (Agar)→ Pre-feeding (Agar, Tryptone)→ Test (Normal Food)** | **-** | **Two-Way ANOVA** |
| **Sup. Figure 8d** | ***Fit*-Gal4/ *slif-anti***<br>***Fit*-Gal4/*TSC1/2*** | **Female and Male** | **Pre-feeding Assay: Starvation (Agar)→ Pre-feeding (Agar, Tryptone)→ Test (Normal Food)** | **-** | **One-Way ANOVA** |

| | | | | | |
|---|---|---|---|---|---|
| **Sup. Figure 9a** | ***Fit*-Gal4/UAS-FIT-HA**<br>***Fit*-Gal4/+;+/UAS-FITΔSP-HA** | **-** | **Western Blot** | **HA, GAPDH** | **-** |
| **Sup. Figure 9b** | **-** | **-** | **Western Blot** | **HA, GAPDH** | **-** |
| **Sup. Figure 9c** | **UAS-FIT /+**<br>***Dilp2*-Gal4/UAS-FIT/+**<br>***Elav*-Gal4/+; UAS-FIT /+**<br>**+/UAS-FITΔSP-HA**<br>***Elav*-Gal4/+; +/UAS-FITΔSP-HA** | **Female** | **Pre-feeding Assay: Starvation (Agar)→ Test (Normal Food)** | **-** | **Unpaired Student *t* test** |
| **Sup. Figure 9d** | ***Elav-GSG/* UAS-FIT** | **Female** | **Pre-feeding Assay: Starvation (Agar)→ Test (Normal Food)** | **-** | **Two-Way ANOVA** |
| **Sup. Figure 9e** | ***Elav*-Gal4/+**<br>***Elav*-Gal4/+;+ /UAS-FIT**<br>***Elav*-Gal4/+;+ / UAS-InR$^{DN}$**<br>***Elav*-Gal4/+;**<br>**UAS-FIT/ UAS-InR$^{DN}$** | **-** | **Two Choice Assay** | **-** | **One-Way ANOVA** |
| **Sup. Figure 10a** | ***w$^{1118}$*** | **Female and Male** | **Overall Mean Intensity of DILP2,**<br>**Total Volume of DILP2 Signal** | **DILP2** | **Two-Way ANOVA** |
| **Sup. Figure 10b** | ***Dilp2*-Gal4/+**<br>***Dilp2*-Gal4/UAS-*Shi$^{ts1}$*** | **Female** | **Overall Mean Intensity of DILP2,**<br>**Total Volume of DILP2 Signal** | **DILP2** | **Two-Way ANOVA** |
| **Sup. Figure 10c** | ***w$^{1118}$***<br>***Fit$^{81}$*** | **Female** | **Overall Mean Intensity of DILP2,**<br>**Total Volume of DILP2 Signal** | **DILP2** | **Two-Way ANOVA** |

| | | | | | |
|---|---|---|---|---|---|
| **Sup. Figure 10d** | ***$w^{1118}$*** | **Female** | **Overall Mean Intensity of DILP2,<br>Total Volume of DILP2 Signal** | **DILP2** | **One-Way ANOVA** |
| **Sup. Figure 10e** | **UAS-FIT /+;+/*Dilp2*-Gal4<br>*Dilp2*-Gal4/+;UAS-FITΔSP-HA /+<br>*Dilp2*-Gal4/UAS-$shi^{ts1}$;+/UAS-FITΔSP-HA<br>*Dilp2*-Gal4/UAS-$shi^{ts1}$; UAS-FIT /+** | **Female** | **Immunohistochemical stain ing** | **DILP2** | **-** |
| **Sup. Figure 10f** | **UAS-FIT /+;+/*Dilp2*-Gal4<br>*Dilp2*-Gal4/+;UAS-FITΔSP-HA /+<br>*Dilp2*-Gal4/UAS-$shi^{ts1}$;+/*UAS-FITΔSP-HA*<br>*Dilp2*-Gal4/UAS-$shi^{ts1}$; *UAS-FIT* /+** | **Female** | **Total Fluorescence Intensity,<br>Overall Mean Intensity of DILP2,<br>Total Volume of DILP2 Signal.** | **DILP2** | **Two-Way ANOVA** |
